# Mucosal B Cells Are Associated with Delayed SIV Acquisition in Vaccinated Female but Not Male Rhesus Macaques Following SIV_mac251_ Rectal Challenge

**DOI:** 10.1371/journal.ppat.1005101

**Published:** 2015-08-12

**Authors:** Iskra Tuero, Venkatramanan Mohanram, Thomas Musich, Leia Miller, Diego A. Vargas-Inchaustegui, Thorsten Demberg, David Venzon, Irene Kalisz, V. S. Kalyanaraman, Ranajit Pal, Maria Grazia Ferrari, Celia LaBranche, David C. Montefiori, Mangala Rao, Monica Vaccari, Genoveffa Franchini, Susan W. Barnett, Marjorie Robert-Guroff

**Affiliations:** 1 Immune Biology of Retroviral Infection Section, Vaccine Branch, National Cancer Institute, National Institutes of Health, Bethesda, Maryland, United States of America; 2 Biostatistics and Data Management Section, National Cancer Institute, National Institutes of Health, Bethesda, Maryland, United States of America; 3 Advanced Bioscience Laboratories, Inc., Rockville, Maryland, United States of America; 4 Duke University Medical Center, Durham, North Carolina, United States of America; 5 USMHRP, Walter Reed Army Institute of Research, Silver Spring, Maryland, United States of America; 6 Animal Models and Retroviral Vaccines Section, Vaccine Branch, National Cancer Institute, National Institutes of Health, Bethesda, Maryland, United States of America; 7 Novartis Vaccines, Cambridge, Massachusetts, United States of America; Emory University, UNITED STATES

## Abstract

Many viral infections, including HIV, exhibit sex-based pathogenic differences. However, few studies have examined vaccine-related sex differences. We compared immunogenicity and protective efficacy of monomeric SIV gp120 with oligomeric SIV gp140 in a pre-clinical rhesus macaque study and explored a subsequent sex bias in vaccine outcome. Each immunization group (16 females, 8 males) was primed twice mucosally with replication-competent Ad-recombinants encoding SIV_smH4_ *env*/*rev*, SIV_239_ *gag* and SIV_239_ *nef*Δ_1–13_ and boosted twice intramuscularly with SIV_mac239_ monomeric gp120 or oligomeric gp140 in MF59 adjuvant. Controls (7 females, 5 males) received empty Ad and MF59. Up to 9 weekly intrarectal challenges with low-dose SIV_mac251_ were administered until macaques became infected. We assessed vaccine-induced binding, neutralizing, and non-neutralizing antibodies, Env-specific memory B cells and plasmablasts/plasma cells (PB/PC) in bone marrow and rectal tissue, mucosal Env-specific antibodies, and Env-specific T-cells. Post-challenge, only one macaque (gp140-immunized) remained uninfected. However, SIV acquisition was significantly delayed in vaccinated females but not males, correlated with Env-specific IgA in rectal secretions, rectal Env-specific memory B cells, and PC in rectal tissue. These results extend previous correlations of mucosal antibodies and memory B cells with protective efficacy. The gp140 regimen was more immunogenic, stimulating elevated gp140 and cyclic V2 binding antibodies, ADCC and ADCP activities, bone marrow Env-specific PB/PC, and rectal gp140-specific IgG. However, immunization with gp120, the form of envelope immunogen used in RV144, the only vaccine trial to show some efficacy, provided more significant acquisition delay. Further over 40 weeks of follow-up, no gp120 immunized macaques met euthanasia criteria in contrast to 7 gp140-immunized and 2 control animals. Although males had higher binding antibodies than females, ADCC and ADCP activities were similar. The complex challenge outcomes may reflect differences in IgG subtypes, Fc glycosylation, Fc-R polymorphisms, and/or the microbiome, key areas for future studies. This first demonstration of a sex-difference in SIV vaccine-induced protection emphasizes the need for sex-balancing in vaccine trials. Our results highlight the importance of mucosal immunity and memory B cells at the SIV exposure site for protection.

## Author Summary

Viral infections can have different disease courses in men and women. Following HIV infection, women generally exhibit lower viral loads and higher CD4 counts than men, but paradoxically progress faster to AIDS. Sex differences result from effects of X-linked genes and hormonal influences, and are believed to be largely based on immune response differences. Nevertheless, little is known about potential sex differences following vaccination. Here we report for the first time a sex bias in response to a SIV vaccine in rhesus macaques, showing that female animals were better protected against acquisition of SIV compared to males. The vaccine-induced immune responses that contributed to this better protection were viral-specific antibodies and immune antibody-secreting B cells, both at the local rectal site of SIV exposure. These results suggest that HIV/SIV vaccines should be better designed to target mucosal exposure sites. Additionally, they indicate that more vaccine studies should include animals of both sexes to address potential differences. Our study also illustrates that inclusion of both sexes can lead to greater complexity in vaccine trial outcomes, necessitating more in depth analyses. However, we believe sex balancing to be particularly important, as approximately 50% of HIV infections worldwide occur in women.

## Introduction

Sex differences in the pathogenesis of numerous viral diseases, including HIV, are well-known [1]. HIV-infected women exhibit lower viral loads and higher CD4 counts than men, but progress faster to AIDS [2]. Women with similar viral loads as men exhibit a 1.6-fold higher risk of AIDS [3]. This sex bias is associated with differences in immune responses. Following viral infections, antigen recognition by pattern recognition receptors, induction of innate and adaptive immune responses, and production of inflammatory cytokines are higher in females than in males [1]. After viral clearance, immune responses in females can remain elevated, contributing to pathogenesis [1]. Less is known regarding sex differences following vaccination. Females have exhibited better immune responses to HSV-2 gD, HBV, and inactivated influenza vaccines [1], but sex-based effects following HIV/SIV vaccinations have not been reported. Using a large number of female rhesus macaques in a pre-clinical SIV vaccine study we uncovered a sex bias in vaccine-elicited immunity and protective efficacy.

Our vaccine strategy is based on mucosally-delivered replicating Ad-recombinants which target myeloid dendritic cells and persist in rectal macrophages, eliciting systemic and mucosal immunity [4]. Following Ad-priming we compared the immunogenicity and protective efficacy of regimens boosted with monomeric SIV gp120 or oligomeric SIV gp140. gp120 immunogens are of interest as they were the form of antigen used as subunit boost for the RV144 clinical trial, the first to show modest protection [5]. Although that vaccine regimen failed to elicit neutralizing antibodies (nAbs) against primary HIV circulating isolates [6], non-neutralizing antibodies exhibiting binding to V1/V2 and high ADCC activity in the presence of low serum IgA levels correlated with reduced infection risk [7–8]. Nevertheless, broadly neutralizing antibodies (bnAbs) are believed important for a highly efficacious vaccine. They develop in a small proportion of HIV-1 patients over prolonged infection, and contribute to maintenance of low viremia [9]. Passive transfer of bnAbs in non-human primates has protected against SHIV infection [10]. Thus, rational design of HIV Env antigens for elicitation of bnAbs is at the forefront of HIV research [11–12]. Native gp140 trimers are thought to be more promising for this purpose compared to monomeric gp120 [13–16] due to the presence of conserved conformational and quaternary epitopes. For example, the potent bnAb 35O22 targets an epitope shared across gp120 and gp41 [17].

The SIV rhesus macaque model is extensively used in pre-clinical vaccine research as SIV transmission and disease progression in macaques resemble human HIV infection [18]. However, SIV monomeric and oligomeric Env immunogens have not been directly compared in this model. We assessed both proteins as booster immunogens, focusing on systemic and mucosal humoral immunity, and evaluating protective efficacy following repeated low-dose SIV rectal challenges. Viremia reductions were modest post-challenge, but we discovered for the first time a sex bias in SIV vaccine outcome. Female but not male macaques exhibited significantly delayed SIV acquisition. These findings are timely in view of recent NIH policy requiring balancing of males and females in animal studies [19]. The mechanisms of acquisition delay point to local mucosal B cell responses.

## Results

### Delayed SIV acquisition following repeated low-dose intrarectal challenges

**Fig 1:**
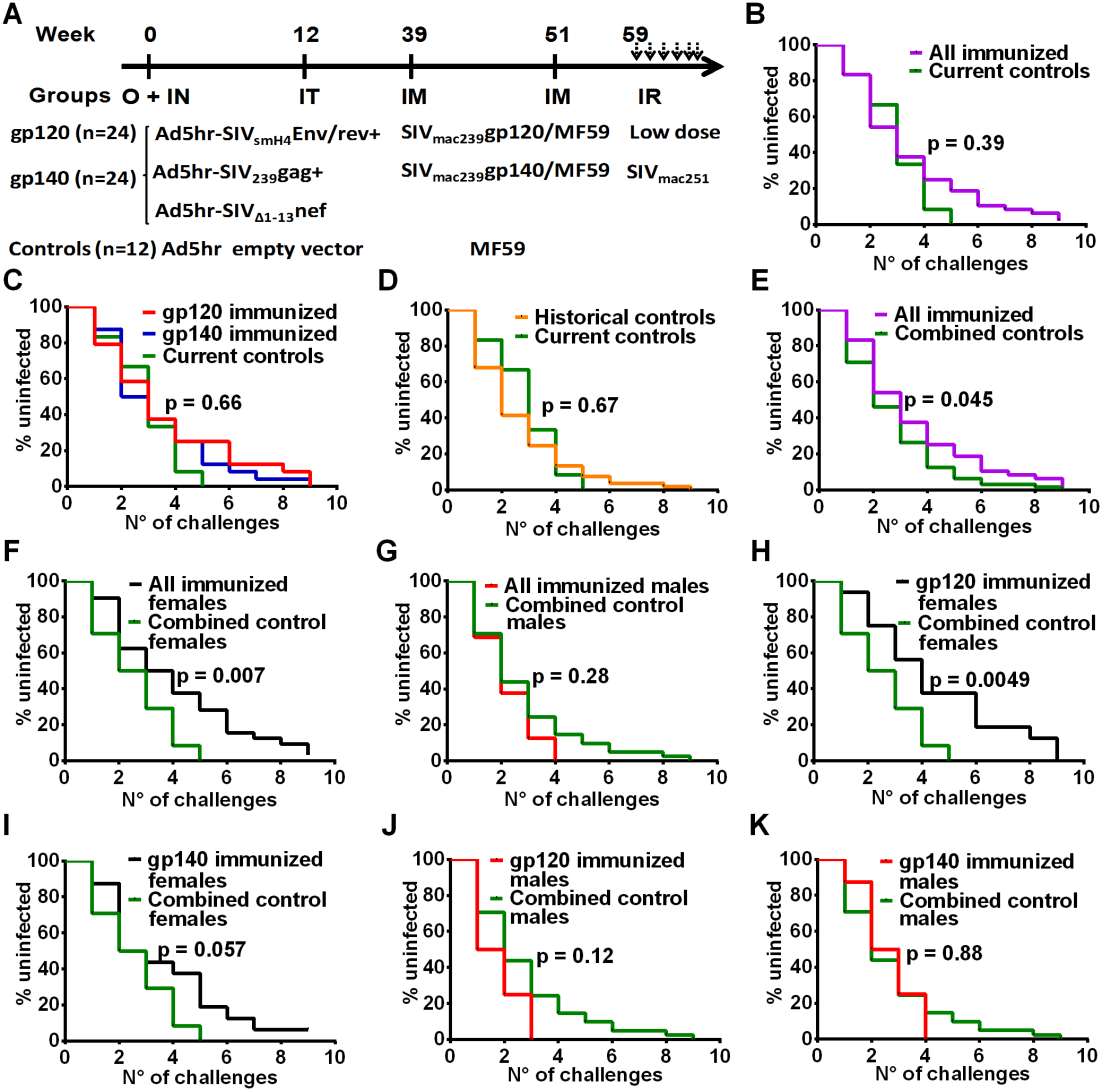
Immunization scheme and delayed acquisition after intrarectal repetitive SIV_mac251_ low dose challenges. (A) Immunization and challenge schedule. O = oral; IN = intranasal; IT = intratracheal; IM = intramuscular; IR = intrarectal. Dosages and further details are provided in on-line Methods. (B) No difference in SIV acquisition between all immunized macaques and current controls (n = 12). (C) No difference in SIV acquisition rate in gp120- and gp140-immunized macaques and current controls. (D) Similar acquisition rate in historical (n = 53) and current controls. (E) Delayed SIV acquisition in all immunized macaques compared to combined controls (n = 65). (F) Delayed SIV acquisition in all immunized females (n = 32) compared to combined control females (n = 24) but not (G) in all immunized males (n = 16) compared to combined control males (n = 41). (H) Delayed SIV acquisition in gp120-immunized females compared to combined control females. (I) Trend for delayed SIV acquisition in gp140-immunized females compared to combined control females. No difference in SIV acquisition rate in gp120-immunized males (J) and gp140-immunized males (K) compared to combined control males.

Macaques mucosally primed with Ad5hr-SIV recombinants and boosted with monomeric gp120 or oligomeric gp140 as described in Materials and Methods and outlined in Fig 1A were challenged intrarectally with repeated low doses of SIV_mac251_ eight weeks following the last immunization. All macaques became infected by the 9^th^ exposure except one gp140-immunized female. No difference in rate of infection was observed between all immunized macaques combined versus the controls (Fig 1B) or between either immunization group and the controls (Fig 1C). As the study included only 12 contemporaneous controls, to achieve greater statistical power we combined these with an additional 53 historical controls (17 females, 36 males) which had been challenged intrarectally at weekly intervals with the same low dose of the same SIV challenge stock. There was no difference in rate of SIV acquisition between the contemporaneous and historical control groups (Fig 1D). A comparison of all the immunized macaques with these combined controls (n = 65) showed a marginally significant difference in rate of SIV acquisition (Fig 1E), suggesting a vaccine effect.

To explore this effect further, we evaluated the rate of SIV acquisition among the immunized male and female macaques and the combined control males and females. We observed significantly delayed acquisition in all the immunized females when compared to the combined control females (Fig 1F), whereas no acquisition delay was seen when all the immunized males were compared to the combined control males (Fig 1G). A direct comparison of all the immunized females versus all the immunized males confirmed a significant acquisition delay in the females (S1A Fig). We next explored the influence of the booster immunogen on acquisition delay. A significant difference was observed when gp120-immunized females were compared to the combined control females (Fig 1H), whereas a marginally non-significant difference was seen when comparing gp140-immunized females versus the combined control females (Fig 1I). In contrast, no delay in acquisition was seen when either gp120- or gp140-immunized males were compared with the combined control males (Fig 1J and 1K). A direct comparison of the gp120- and gp140-immunized females versus the similarly immunized males again confirmed the delayed acquisition in gp120- but not gp140-immunized females (S1B and S1C Fig). Overall the delay in SIV acquisition of the gp120 immunized females was clearly a vaccine effect and provides the first demonstration of a sex bias in SIV vaccination outcome.

### Immunogenicity of monomeric gp120 and oligomeric gp140

To understand the basis for the significantly delayed acquisition observed in gp120 immunized but not gp140 immunized females, we conducted a thorough analysis of systemic and mucosal humoral immune responses throughout the course of immunization and post-challenge. We first compared the two immunization groups. Oligomeric gp140 proved to be more immunogenic than gp120 as summarized in Tables 1 and 2 and detailed in the accompanying supplemental figures. Systemic Env-specific binding antibodies following Ad5hr-recombinant immunizations (wk 14) were boosted to titers over 10^6^ (wk 53) in both immunization groups (S2A–S2C Fig). The gp120 group exhibited similar antibody titers against gp140 and gp120 but gp140-immunized animals developed higher titers to gp140 with an overall higher titer to gp140 compared to gp120-immunized macaques (Table 1; S2A–S2C Fig). Antibody levels were maintained between wk 53 post-vaccination and 2 weeks post-infection (2wkpi) in both groups. The gp140 immunized macaques also developed higher cyclic V2-specific binding antibody titers than the gp120 group (Table 1; S2D Fig).

**Table 1:** Comparative immunogenicity of monomeric gp120 and oligomeric gp140: serum binding and functional antibody activities.

	gp120 immunized		gp140 immunized		gp120 vs gp140
Serum Antibody Responses	geometric mean	95% CL	geometric mean	95% CL	p value
Binding titer to gp120	2.18x10^6^	(1.38x10^6^, 3.42x10^6^)	2.06x10^6^	(1.38x10^6^, 3.07x10^6^)	0.90
Binding titer to gp140	2.29x10^6^	(1.54x10^6^, 3.41x10^6^)	4.51x10^6^	(3.28x10^6^, 6.22x10^6^)	**0.0059**
Binding titer to cyclic V2	8.13x10^4^	(6.50x10^4^, 1.02x10^5^)	1.12x10^5^	(8.84x10^4^, 1.41x10^5^)	**0.034**
Neutralizing titer^a^	5.97x10^5^	(3.42x10^5^, 1.04x10^6^)	3.74x10^5^	(2.29x10^5^, 6.11x10^5^)	0.12
ADCC to gp120 targets^b^	6.00x10^4^	(4.03x10^4^, 8.92x10^4^)	2.81x10^5^	(1.66x10^5^, 4.77x10^5^)	**<0.0001**
ADCC to gp140 targets^b^	3.74x10^5^	(2.57x10^5^, 5.44x10^5^)	1.78x10^6^	(1.26x10^6^, 2.52x10^6^)	**<0.0001**
	**mean**	**± SEM**	**mean**	**± SEM**	**p value**
Phagocytosis of gp120 beads^c^	1.12	± 0.01	1.21	± 0.01	**<0.0001**
Phagocytosis of gp140 beads^c^	1.25	± 0.01	1.27	± 0.01	0.59

Immune responses were evaluated on serum samples obtained at week 53, 2 weeks following the second Env protein immunization.

^a^Neutralization of tier 1 SIV_mac251.6_;

^b^ADCC expressed as 50% maximum killing titer;

^c^Phagocytic score/background phagocytic score. CL = confidence limits.

Serum nAb titers against tier 1 SIV_mac251.6_ were comparable in both immunization groups (Table 1; S3A Fig). No neutralization of challenge-related tier 3 SIV_mac251.30_ developed. Higher ADCC activity (S3B and S3C Fig) was elicited by gp140 compared to gp120 immunization, regardless of whether gp140- or gp120-coated targets were tested (Table 1). Similarly, antibody-mediated phagocytosis of gp140-coated beads pre-and post-challenge was elevated in both immunization groups compared to controls (p<0.0001; S3D Fig). gp140-immunized macaques phagocytosed gp120-coated beads significantly above control and gp120-immunized macaque levels (wk 53, Table 1; S3E Fig), whereas phagocytosis by gp120-immunized macaques was higher than that of gp140-immunized macaques 2wkpi (p = 0.0034; S3D Fig).

Mucosal binding antibodies were also assessed during the immunization regimen. Rectal gp120- and gp140-specific IgA and IgG were elicited following mucosal Ad-recombinant priming (wk 14) in both immunization groups. After systemic Env immunization, mucosal IgG was significantly boosted (wk 53) while Env-specific IgA was maintained at post-Ad levels (S4A–S4D Fig). In most cases, IgA and IgG mucosal antibodies in both groups showed elevated reactivity to gp120 at wk 53. gp140-immunized animals developed higher levels of Env-specific rectal IgG against gp140 (wk 53) compared to gp120-immunized macaques (Table 2).

**Table 2:** Comparative immunogenicity of monomeric gp120 and oligomeric gp140: mucosal and bone marrow responses.

Mucosal and Bone Marrow Responses	gp120 immunized		gp140 immunized		gp120 vs gp140
Mucosal Antibody Responses	Mean	± SEM	Mean	± SEM	p value
Rectal gp120-specific IgA^a^	0.21	± 0.066	0.20	± 0.092	0.19
Rectal gp140-specific IgA^a^	0.043	± 0.026	0.18	± 0.13	0.055
Rectal gp120-specific IgG^a^	39.51	± 7.14	53.15	± 8.05	0.12
Rectal gp140-specific IgG^a^	12.53	± 1.71	27.32	± 3.15	**0.0002**
**Bone Marrow Responses**					
Env-specific IgA memory B cells^b^	6.55	± 1.94	5.49	± 1.24	0.58
Env-specific IgA PB/PC^b^	0.80	± 0.26	1.85	± 0.48	**0.033**
Env-specific IgG memory B cells^b^	2.30	± 0.40	3.20	± 0.58	0.22
Env-specific IgG PB/PC^b^	1.39	± 0.34	2.37	± 0.42	**0.013**

Immune responses were evaluated on mucosal secretions and bone marrow samples obtained at week 53, 2 weeks following the second Env protein immunization.

^a^ng specific/μg total.

^b^Percent Env-specific ASC relative to total ASC in bone marrow.

Bone marrow (BM) antibody secreting cells (ASC) were next assessed by ELISpot. SIV Env-specific IgG and IgA memory B cells significantly declined after peak elicitation (wk 53), but rebounded 2wkpi (S5A and S5B Fig). IgA memory B cells developed at higher levels than IgG memory B cells at wk 53 in both groups (p = 0.0049 and 0.036), and also 2wkpi (p = 0.0001 and 0.023) (S5C and S5D Fig). Env-specific plasmablasts (PB) and plasma cells (PC) exhibited a similar response pattern as memory B cells, but displayed smaller IgG and IgA ASC declines between wks 53 and 57 as expected for long-term memory cells (S6A and S6B Fig). The gp140 group maintained higher levels of both IgG and IgA PB/PC prior to challenge (wk 53, Table 2; wk 57, S6A and S6B Fig). In both groups IgG PB/PC were elevated compared to IgA PB/PC at wk 53 (p = 0.0072 and 0.014, respectively), but IgA was higher than IgG in gp120-immunized macaques 2wkpi (p = 0.023; S6C and S6D Fig).

With regard to cellular immune responses, we investigated SIV specific CD4^+^_TM_ and CD8^+^_TM_ T-cell responses in PBMC 2wkpi. SIV_smH4_ Env-specific CD4^+^ and CD8^+^ T-cell responses, representative of *env* encoded in the Ad-recombinant, were comparable between immunization groups, and appeared post-infection in controls (S7A and S7C Fig). In contrast, gp140- compared to gp120-immunized macaques exhibited a trend of elevated CD4^+^ and CD8^+^ T-cell responses following SIV_mac239_ Env stimulation, suggesting a more effective booster immunization (S7B and S7D Fig). Similar results were seen after summing responses to Env, Gag, and Nef (S7E–S7H Fig).

### Sex-related difference in immune responses to monomeric gp120 and oligomeric gp140

**Fig 2:**
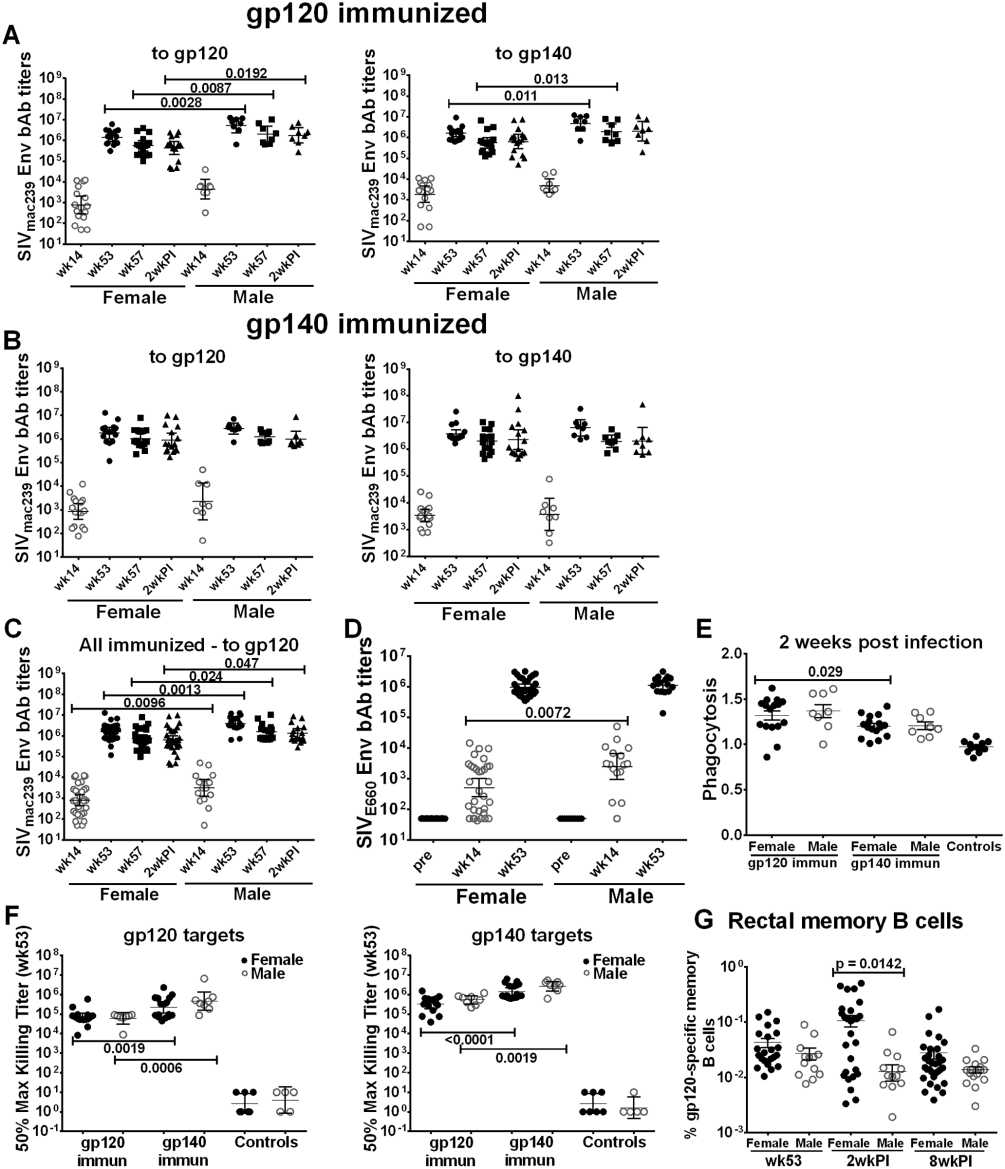
Comparison of immune responses between female and male macaques. Binding antibody titers to SIV_mac239_ gp120 and gp140 over the course of immunization and following infection in (A) gp120- and (B) gp140-immunized female and male macaques. (C) Binding antibody titers by sex of combined gp120- and gp140 immunization groups to SIV_mac239_ gp120 over the course of immunization and 2wkpi. (D) Binding antibody titers to SIV_E660_ gp120 over the course of immunization in females and males of combined gp120- and gp140-immunization groups. Pre-bleed samples were not tested but binding titers of control macaque samples at all time points were <50. Titers are expressed as geometric means with 95% CL. (E) Serum phagocytic activity (phagocytosis score/background phagocytosis) to gp140 targets 2wkpi in females and males of the gp120- and gp140 immunization groups. (F) Serum ADCC activity of female and male macaques to gp120 and gp140 targets by immunization group at wk 53. (G) Rectal gp120-specific memory B cells (identified by flow cytometry) in female and male macaques of combined gp120- and gp140-immunization groups at 2 wk post-second Env boost (wk 53), 2wkpi, and 8wkpi. Mean values ± SEM are shown in E and G. One gp140-immunized macaque remained uninfected, and is omitted from 2wkpi analyses. In panel G, rectal samples of 15 macaques (6 from each gp120- and gp140-immunization group and 3 controls) are not shown at wk 53 as samples were lost due to a processing error.

Having shown that immune responses in general were elevated in the gp140 immunized macaques, but that SIV acquisition delay was observed in gp120 immunized female macaques, we next analyzed these data by sex. Systemic binding antibodies to the SIV_mac239_ Env boosting immunogens were higher in gp120-immunized males compared to females against both gp120 and gp140 targets prior to challenge (wks 53 and 57), and were maintained at higher levels against gp120 2wkpi (Fig 2A). A similar result was not seen in the gp140-immunized animals (Fig 2B). Males of both groups combined exhibited higher titers to gp120 than females at all time points (Fig 2C). Antibody responses to SIV Env_E660,_ representative of SIV Env_smH4_ in the Ad-recombinant, were higher in immunized males compared to females following priming (wk 14; Fig 2D). However, no significant sex differences were seen in neutralizing antibody titers or binding titers to cyclic V2 (S8A–S8C Fig); BM ASC (S9A–S9D Fig), or rectal Env-specific IgA and IgG (S10A–S10D Fig). Although no significant sex difference by group was observed in phagocytic activity, gp120-immunized females maintained higher activity against gp140-coated beads compared to the gp140 group (Fig 2E). Additionally, no sex differences were seen in ADCC activity by group; however, consistent with results of the group analysis (S3B and S3C Fig) gp140-immunized females and males maintained higher activity against both gp120 and gp140 targets (Fig 2F). Female macaques displayed significantly higher rectal Env-specific memory B cell levels than males 2wkpi (Fig 2G), regardless of immunization group. A similar trend was seen both prior to challenge (wk 53) and 8wkpi.

Env-specific CD4^+^_TM_ and CD8^+^_TM_ T-cell responses showed no sex-based differences (S11A–S11D Fig), although females tended to exhibit higher responses following Env_239_ stimulation, indicative of the protein boosts derived from that strain (S11B and S11D Fig). When CD4^+^_TM_ and CD8^+^_TM_ responses against Env, Gag, and Nef were summed, results were similar in animals stimulated with Env_smH4_ peptides, matched to the *env* gene in the Ad-recombinant, (S11E and S11G Fig) whereas females showed higher CD4^+^_TM_ and CD8^+^_TM_ T-cell responses than males in the animals stimulated with Env_239_ peptides, matched to the Env booster immunogens, significantly so for the CD4 responses (p = 0.019; S11F and S11H Fig).

### Immunological correlates of delayed SIV acquisition

**Fig 3:**
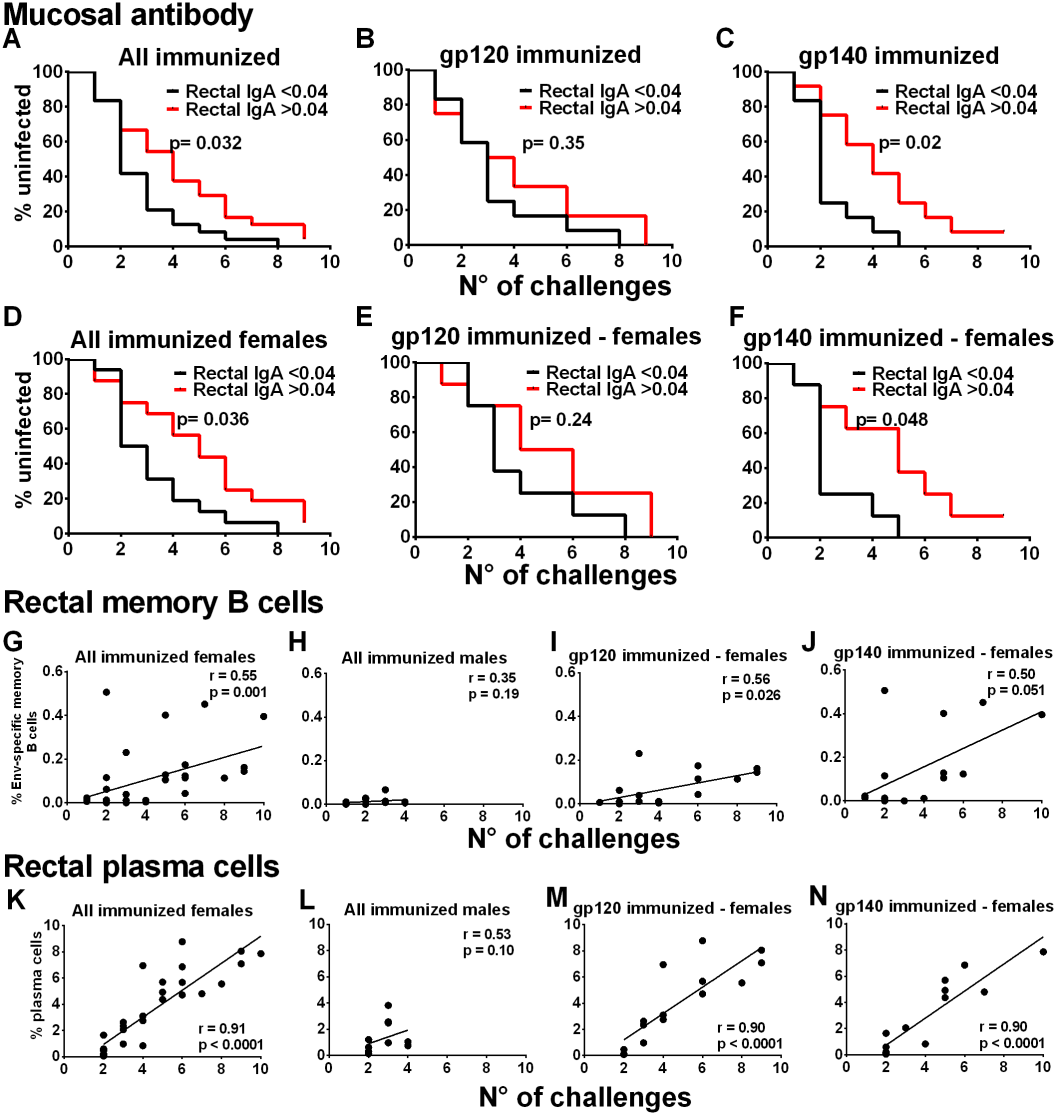
Immunological correlates of delayed SIV acquisition. Influence of rectal Env-specific IgA at wk 55 on the rate of infection in (A) all immunized macaques, (B) in gp120 immunized macaques, and (C) in gp140 immunized macaques. Influence of rectal Env-specific IgA at wk 55 on the rate of infection in (D) all immunized females, (E) in gp120-immunized females, and (F) in gp140-immunized females. gp120 and gp140-immunized macaques were tested against gp120 and gp140 proteins respectively. Control background levels were subtracted prior to analysis. Correlation analysis of Env-specific memory B cells in rectal tissue identified by flow cytometry 2wkpi with number of challenges to become infected in (G) all immunized females, (H) all immunized males, (I) gp120-immunized females, and (J) gp140-immunized females. Correlation analysis of rectal plasma cells identified by flow cytometry 2wkpi with number of challenges to become infected in (K) all immunized females, (L) all immunized males, (M) gp120-immunized females, and (N) gp140-immunized females. One gp140-immunized female remained uninfected and is excluded from the 2wkpi time point in panels G, J, K, and N. In panels K-N, PC were identified following the first challenge using IRF-4 and BCL2 intracellular markers. Subsequently, all data were obtained using the surface marker CD138 and IRF-4. As the two approaches are not comparable, the week one challenge data are omitted.

Analysis of all the immunogenicity data showed that neither humoral nor cellular systemic immune responses, including serum binding antibodies, serum neutralizing or non-neutralizing activities, bone marrow memory B cells and PB/PC, and CD4^+^ and CD8^+^ T cell responses, correlated with SIV acquisition delay. With regard to mucosal immune responses, Env specific IgG in rectal secretions was not associated with acquisition delay in either gp120- or gp140-immunized male or female macaques (S12 Fig). However, although present at lower levels, Env-specific IgA in rectal secretions significantly correlated with delayed acquisition (Fig 3A). All immunized animals with rectal Env-specific IgA levels above the median (0.04ng/μg total IgA) required more SIV exposures for infection. The difference remained significant in the gp140 group alone (tested against gp140, Fig 3C) but not in the gp120 group (tested against gp120, Fig 3B). This same pattern was exhibited by immunized females. Higher Env-specific rectal IgA levels in all immunized females and in gp140-immunized females but not in gp120-immunized females were associated with an increased number of challenges (Fig 3D–3F). Env-specific rectal IgA in vaccinated males did not correlate with delayed acquisition (S13 Fig). As delayed acquisition of immunized females was most evident in gp120-immunized macaques (Fig 1H and 1I), additional factors must have been involved.

To pursue the role of mucosal immunity in delayed acquisition, we next examined Env-specific memory B cells and total PB and PC in rectal tissue by flow cytometry [20–21] (S14 Fig **)**. Consistent with the higher rectal Env-specific memory B cell levels 2wkpi in immunized females compared to males (Fig 2G), the rectal Env-specific memory B cell levels 2wkpi were significantly correlated with challenge exposures in all immunized females, but not males (Fig 3G and 3H). This correlation remained significant in gp120-immunized females and approached significance in gp140-immunized females (Fig 3I and 3J). Total rectal PC levels were significantly correlated with acquisition delay in all immunized females but not males (Fig 3K and 3L) and in gp120- and gp140-immunized females analyzed separately (Fig 3M and 3N). Overall, our data strongly implicate a local mucosal B cell contribution in delayed acquisition of vaccinated female macaques.

### Reduced acute viremia following repeated low-dose SIVmac251 rectal challenges

**Fig 4:**
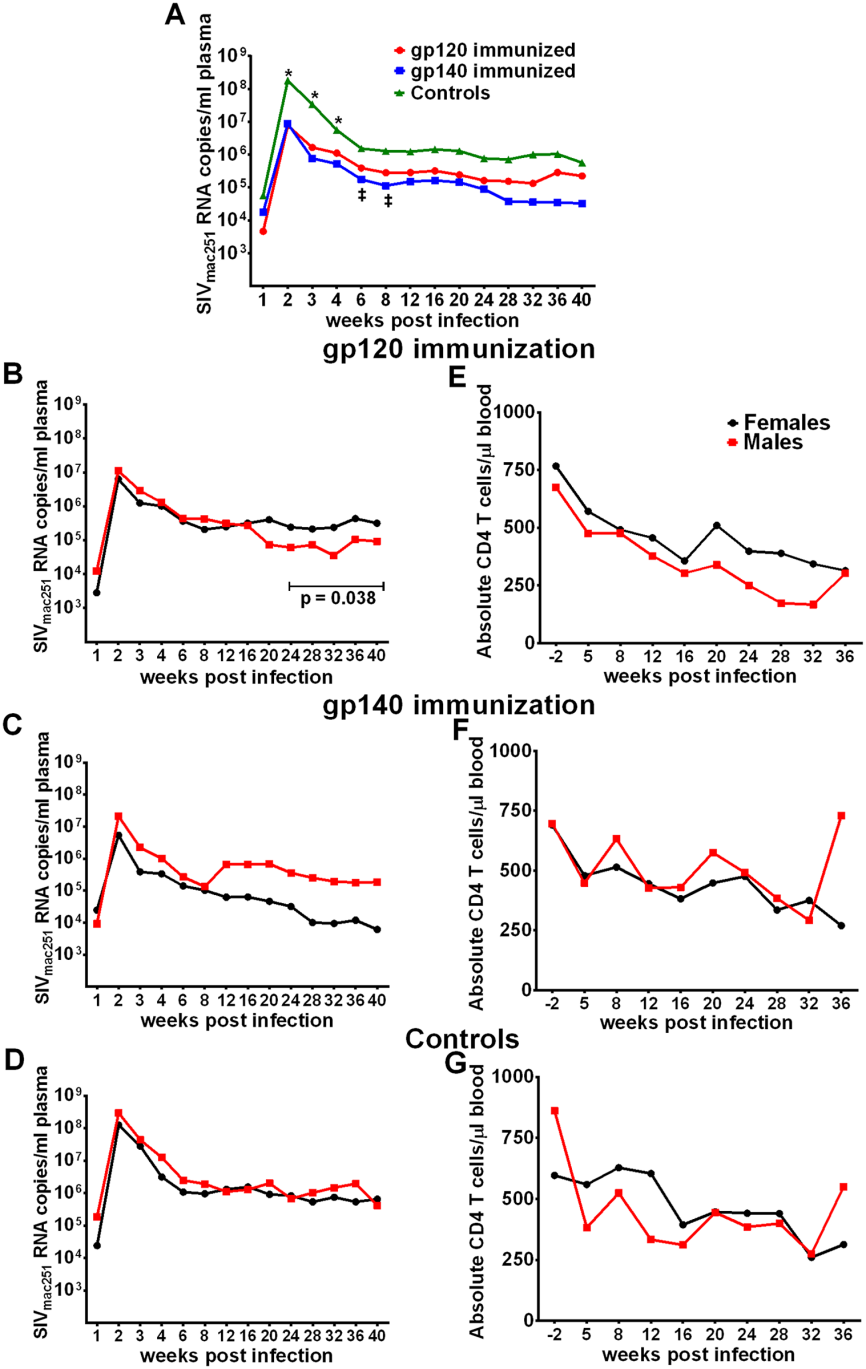
Viral loads post-infection by immunization group and sex. (A) Geometric mean plasma viral loads by immunization group. (B-D) Geometric mean plasma viral loads and (E-G) mean CD4 T cell counts in males and females in gp120, gp140, and control macaque groups.

A secondary outcome of this study was modestly reduced acute phase viremia in the immunized macaques compared to the controls. Median peak viremia for the gp120 and gp140 groups (1.79x10^7^ and 2.16x10^7^ SIV RNA copies/ml, respectively) were reduced nearly one log compared to controls (1.71x10^8^ SIV RNA copies/ml; p<0.05). Viremia differences between gp120- and gp140-immunized macaques and controls were significant at 2, 3 and 4wkpi, while the gp140 group also exhibited lower viremia at 6 and 8wkpi (Fig 4A). Viral loads of the individual macaques are shown in S15 Fig.

In contrast to SIV acquisition, no sex bias was observed in viremia reduction. Both females and males of both immunization groups as well as the controls exhibited similar viral loads during the acute phase of infection (Fig 4B–4D). Similarly, CD4 counts over the period of follow-up were similar between the sexes (Fig 4E–4G). We did observe a decrease in viral loads of males compared to females in the gp120 group over weeks 24 to 40 post infection (Fig 4B). A similar difference was not seen in the gp140 immunized macaques, however, we cannot reach a firm conclusion regarding an immunization group difference as a number of macaques in the gp140 group had been euthanized prior to 40 weeks of follow up (see below). Viral loads during the acute phase of infection for the historical controls were available for 41 of the additional 53 macaques, however, no acute viral load difference was observed between the current and historical controls or between males and females of the combined current and historical control groups (S16 Fig).

**Fig 5:**
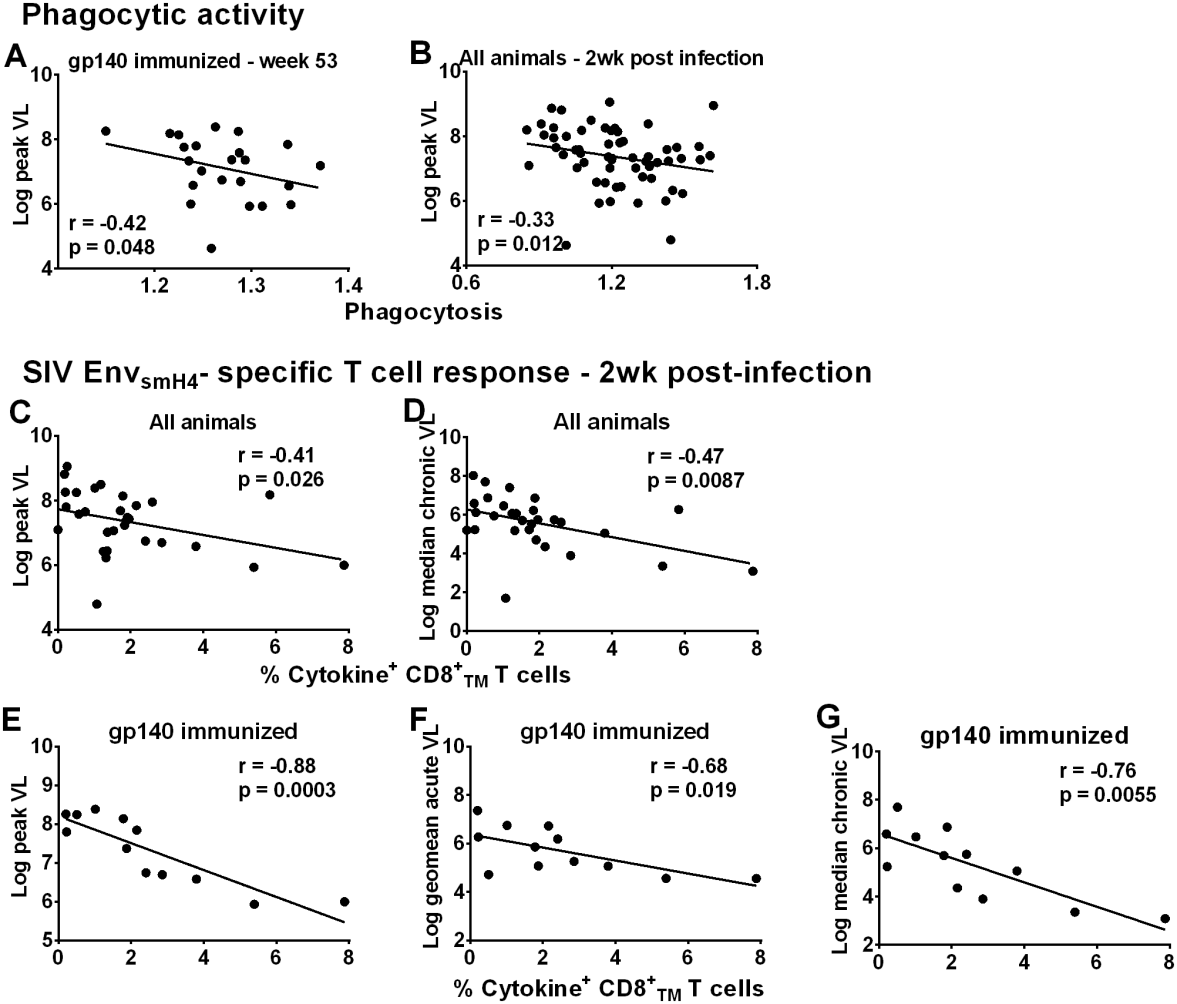
Immunological correlates of viremia control. Correlation of phagocytic activity (expressed as phagocytosis score/background phagocytosis) of gp140-immunized macaques using gp140 targets 2 weeks post-second Env boost (wk 53) with peak viral load (A). Correlation of phagocytic activity of all macaques 2wkpi with peak viral load (B). Correlation of peripheral Env_smH4_-specific CD8^+^_TM_T cells (% CD8^+^_TM_ T cells expressing IL-2, IFN-γ, and TNF-α) in all animals with reduced peak viral load (C) and chronic viremia (median over weeks 8–24) (D). Correlation of Env_smH4_-specific CD8^+^_TM_ T cells (% CD8^+^_TM_ T cells expressing IL-2, IFN-γ, and TNF-α) 2wkpi in gp140-immunized macaques with (E) peak viral load, (F) acute viral load (geometric mean over weeks 1–6), and (G) chronic viremia (median over weeks 8–24). One gp140-immunized female macaque remained uninfected so is not included in these correlations with viral load. Due to the large number of macaques only half of the macaques in each group were assessed for CD8^+^_TM_ T cell SIV_smH4_ Env-specificity.

We next examined vaccine-induced immune responses associated with the modestly reduced acute phase viremia in the immunized macaques. We found that phagocytic activity prior to challenge (wk 53) against gp140 targets by the gp140-immunization group, which displayed more prolonged viremia control than the gp120-immunization group (Fig 4A), was significantly correlated with reduced viremia (Fig 5A). Phagocytosis by all macaques was inversely correlated with peak viremia 2wkpi (Fig 5B). No correlation with neutralizing antibody or ADCC activity was observed (S17 Fig).

CD8^+^ T-cell responses contribute to viremia control in natural infection [22–23], confirmed in numerous pre-clinical vaccine studies [24–32]. SIV_smH4_ Env-specific CD8^+^_TM_ T-cells in all macaques significantly correlated with reduced peak and chronic viremia (Fig 5C and 5D). By immunization group, a significant inverse correlation was only observed between SIV_smH4_ Env-specific cytokine-producing CD8^+^_TM_ T-cells of gp140-immunized macaques and viremia levels at peak and acute-phase time points and during the chronic phase of infection (Fig 5E–5G). No correlations with SIV_mac239_ Env-specific CD8+TM T cells were observed. Overall, the contribution of cellular immunity to viremia reduction was most evident in gp140-immunized macaques.

Although cellular responses in macaques overall and in gp140-immunized macaques were associated with better viremia control (Fig 5C–5G), this outcome was not reproduced in females. SIV_smH4_ Env-specific CD8^+^_TM_ responses in all males but not all females correlated significantly with reduced peak, acute-phase, and chronic viremia (p = 0.0055, 0.0004, and 0.0086, respectively; S18A and S18B Fig).

### Additional post-infection outcomes

**Fig 6:**
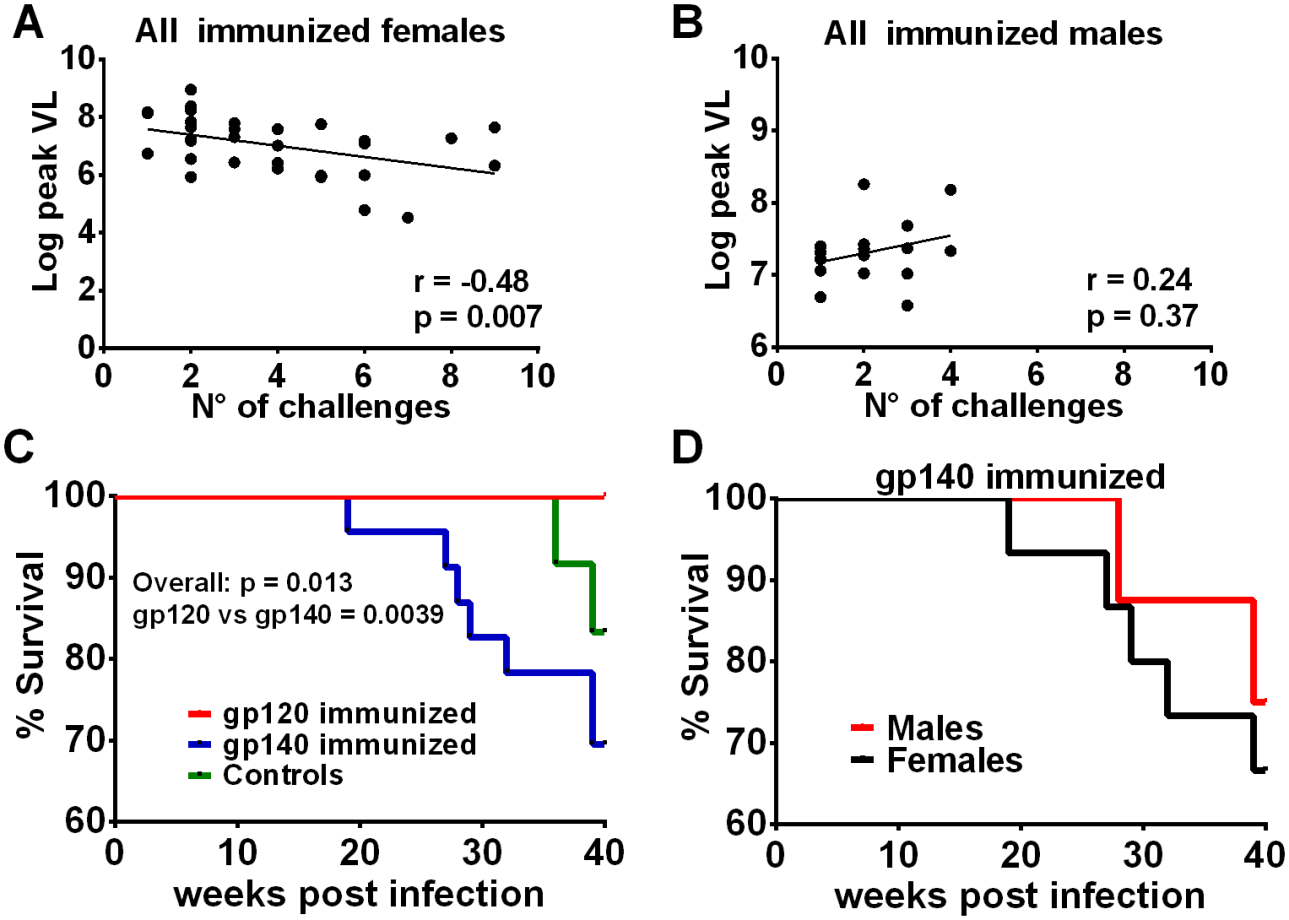
Additional post-infection outcomes by immunization group and sex. Correlation of the number of challenges required to become infected with peak viremia in all immunized females (A) but not in males (B). (C) Comparison of survival for gp120- and gp140-immunized macaques and control macaques. The overall p value was obtained by a logrank test. All 24 macaques in the gp120 immunization group survived at least 40 weeks, 7 of the 23 infected macaques in the gp140 group were euthanized between weeks 19 and 39, and 2 of 12 controls were euthanized between weeks 36 and 39. (D) Survival of female and male macaques in the gp140 group. Five of the 15 infected females were euthanized between weeks 19 and 39; 2 of 8 males were euthanized between weeks 28 and 39.

We observed that the number of challenges necessary to infect immunized females but not males correlated inversely with peak viremia (Fig 6A and 6B). Thus we speculate that repeated exposures boosted immunity, leading to better acute viremia control. Over 40 weeks of follow-up, no group differences were seen in males or females with regard to viral loads (S19A and S19B Fig) or CD4 counts (S19C and S19D Fig), with the exception as mentioned above, that gp120 immunized females exhibited higher viral loads than similarly immunized males over weeks 24–40 of the chronic phase (Fig 4B). However, in spite of enhanced immunogenicity, significantly more gp140-immunized macaques (*n* = 7) met established criteria and had to be euthanized before 40wkpi compared to gp120-immunized macaques (*n* = 0) and the controls (*n* = 2) (Fig 6C). While 5 females and 2 males in the gp140 group were euthanized before 40wkpi (Fig 6D) this difference was not statistically significant.

## Discussion

Here we report for the first time a sex bias in SIV vaccine-induced protective efficacy. Delayed SIV acquisition in females was associated with local B cell immunity, including Env-specific mucosal IgA, Env-specific rectal memory B cells, and rectal PC. Our results highlight the importance of mucosal immunity and development of memory B cells at the site of viral exposure for an effective vaccine.

The correlations of anamnestic Env-specific rectal memory B cell and total rectal PC responses with acquisition delay were obtained with samples obtained 2wkpi. It is possible that these B cell responses could have been boosted by the series of repeated low dose viral exposures necessary to infect the female macaques. However, in the absence of any detectable infection over the course of these weekly challenges, these responses, initially elicited by vaccination, even if boosted, were contributing to protective efficacy. Similar responses were not observed in control macaques. Future studies should investigate more fully the possibility of antigenic boosting by repeated low-dose challenge exposures.

Our previous report of vaccine-induced rectal IgA correlating with delayed SIV_mac251_ acquisition [33] is confirmed here and extended by demonstrating the sex bias. Other reports have also associated mucosal antibody with protection. Vaccine-induced rectal antibodies mediating transcytosis correlated with decreased chronic viremia [34]. Macaques protected against repeated vaginal SHIV challenges exhibited vaginal IgAs that blocked transcytosis and vaginal IgGs with neutralizing and/or ADCC activity [35]. Following intravenous SIV_mac251_ challenge, aerosol-vaccinated macaques exhibited reduced CD4^+^ T-cell depletion in the lung correlated with viral-specific IgA in bronchoalveolar lavage and nasal fluid [36]. Thus the rationale for continued study of mucosal antibodies in vaccine efficacy is well-substantiated.

We previously reported a correlation of vaccine-elicited HIV and SIV Env-specific IgG and IgA peripheral blood memory B cells with reduced viremia [37]. Here we extend this finding, demonstrating the importance of Env-specific memory B cells and PC at the mucosal exposure site for delayed SIV_mac251_ acquisition. It will be important to further explore how vaccine designs can foster homing of memory B cells to the mucosa and enhance their retention. Here we believe the replicating Ad-recombinants played a major role. We have previously shown that the biodistribution of this vector following administration to the upper respiratory tract is broad, and that it exhibits persistent expression in rectal macrophages [4]. Certainly, continued exploration of vaccine-elicited mucosal immune responses in males and females is warranted, along with pursuit of vaccine regimens that target the intestinal mucosa.

Females exhibited a higher percentage of SIV Env-specific memory B cells in rectal tissue, consistent with higher basal immunoglobulin levels and greater humoral responses to antigens in women compared to men [1]. While mucosal antibodies correlated with significant acquisition delay in females, male macaques exhibited higher serum antibody binding titers than females at the time of peak response, 2 weeks after the second envelope boost. Nevertheless, no sex bias was seen in neutralizing or non-neutralizing antibody activities. The proportion of IgG subtypes in males versus females should be examined, as IgG3 V1V2-specific antibodies that mediate ADCC correlated with decreased risk of HIV infection in the RV144 trial, but exhibited a short half-life [38]. Recent development of reagents for use in subtyping macaque IgG should allow this question to be addressed.

Additionally, Fc-receptor differences may exist between males and females. Polymorphisms in IgG Fc-receptors modulate antibody binding affinity for IgG subtypes, and affect antibody-dependent functions [39–40]. Moreover, differences in Fc glycosylation can affect antibody function [41]. Fucosylation modulates IgG1 binding to FcγRIIIa [42]. In the absence of fucose, binding is enhanced, resulting in improved ADCC activity [43]. A non-fucoslylated variant of bNAb 2G12 exhibited greater ADCVI activity against HIV and SHIV isolates [44]. Fc glycosylation differences also modulate ADCP activity [45]. Further, Fc agalactosylation and asialyation have been associated with better HIV control [46]. Differences in Fc glycosylation patterns between males and females have been established [47] and could have impacted our results.

Delayed SIV acquisition in immunized females was greatest in gp120-immunized rather than gp140-immunized macaques that exhibited enhanced humoral immunity. Moreover, gp140-immunized animals met criteria for euthanasia earlier than gp120-immunized macaques although a sex bias was not observed. Although not excluding investigations of oligomeric gp140, this result validates continued study of gp120, the form of immunogen used in the RV144 trial, as a vaccine immunogen. The basis for the different outcome in gp120 immunized macaques while gp140 immunization appeared more immunogenic, however, is not known. Differences in antibody epitope specificities elicited by the different immunogens as well as IgG subtypes and Fc/Fc-R differences as discussed above might explain these outcomes. It may also be the case that higher antibody titers are not beneficial. This has been seen in other infectious diseases. For example, high titers and avidity of vaccine-elicited non-neutralizing antibodies against influenza have been associated with development of more severe disease [48]. Moreover, some non-neutralizing antibodies may be detrimental to protective efficacy. In the RV144 trial, V1V2-specific antibodies that mediate ADCC correlated with protection against acquisition, however high serum Env-specific IgA correlated with infection risk, possibly blocking protective ADCC responses [49]. We did not examine serum Env-specific IgA levels, but they warrant evaluation. Antibody-dependent enhancement of infection can also occur via complement and Fc receptors, dependent on antibody titer and receptor affinity [50]. Both FcγRIIa and FcγRIIIa receptor genetic polymorphisms increase receptor avidity for immune complexes [40]. Notably, the FcγRIIIa genotype was associated with HIV infection rate in the VAX004 trial [51]. Thus, genotyping receptors in females and males may also help explain our complex results.

Viral loads exhibited in this study not unexpectedly inversely correlated with CD8^+^ T-cell responses. By immunization group, a significant correlation of these cellular responses with reduced viremia was only seen in gp140 immunized animals, perhaps due to additional epitopes present in gp140. The gp140 immunized macaques also exhibited more persistent acute viremia reductions. It is possible that these cellular immune responses initially contributed to stronger acute viremia control in this immunization group while at the same time enhanced humoral immunity led to later detrimental effects as suggested above, resulting in the gp140 immunized macaques meeting euthanasia criteria earlier than the gp120 immunized animals. In this regard, significant correlations of CD8^+^ T cell responses with decreased viremia were exhibited in all male but not female macaques (S18 Fig), perhaps reflecting a greater waning of vaccine-induced CD8^+^ T-cell responses during infection delay in females. This might have abbreviated the period of time during which the CD8 T cells were able to effectively control viremia. Among humoral responses, phagocytic activity 2wkpi correlated with decreased viremia in all macaques, but a significant correlation of ADCP prior to infection (wk 53) was only present in gp140-immmunized macaques, a result possibly influenced by antibody quality as discussed above.

The sex bias in immunity [52], especially mucosal immunity [53–54], is profound and can be attributed to both hormonal influences and contributions of X-linked genes. The microbiome plays a major role in shaping mucosal immune responses [55] and can impact mucosal infections. Steroid hormones can also modulate the microbiome, leading to distinct sex profiles [56]. Overall the microbiome composition is critical in HIV transmission and pathogenesis, can influence HIV acquisition [57], and is a key area for further investigation of the sex bias in SIV acquisition.

Female sex hormone changes throughout the menstrual cycle impact susceptibility to vaginal HIV infection by affecting all arms of the immune system. A “window of vulnerability” in the late secretory phase of the cycle during which risk of sexually transmitted infections is highest was postulated [58], and corroborated by the demonstrations during the secretory phase of more frequent vaginal SHIV transmission to macaques [59] and better *ex vivo* HIV infection of human cervical explants [60]. We did not synchronize our female macaques, as rectal challenges were planned. However, fluctuations in female sex hormone levels could affect HIV/SIV acquisition by other than vaginal routes of exposure. Estrogen receptors (ER) are expressed by cells in a variety of tissues in addition to the reproductive tract. ERα is expressed in T and B lymphocytes, dendritic cells, macrophages, monocytes, natural killer cells and mast cells [61], and influences intestinal levels of proinflammatory cytokines, including TNFα [62]. An examination of gut biopsies from men and women directly demonstrated that women have higher levels of immune activation and inflammation compared to men [53]. The profound effects of ERα on DC development and function greatly influence the quality of adaptive immune responses. ERβ is expressed predominantly in the brain, cardiovascular system, and colon and is found mainly on epithelial cells [63]. It plays an important role in cellular differentiation and maintenance of cellular homeostasis in the colon [64]. In addition, by suppressing chloride ion secretion across the colonic epithelium, estrogen controls fluid retention during different stages of the menstrual cycle [65]. Estrogen also increases mucin content of the protective mucus layer in the intestine and increases mucus viscosity and elasticity [66]. ERα and ERβ play different roles in controlling B cell maturation and selection. Engagement of both by estrogen can alter B cell maturation, whereas triggering of ERα influences development of autoimmunity [67]. In rhesus macaques the frequency of ASC in not only genital mucosal but also systemic lymphoid tissues, bone marrow, and PBMC exhibited profound changes throughout the menstrual cycle [68]. Overall, little is known regarding the influence of female sex hormones on other than vaginal viral exposures, however, as illustrated above, these hormones affect innate and adaptive immune responses, intestinal homeostasis and integrity, biophysical properties of protective mucus, and immune activation and inflammation in more than just reproductive tissue. Thus, it is reasonable to take into account potential hormonal effects in future vaccine studies.

Our results showing a clear sex bias in vaccine challenge outcome correlated with local mucosal humoral immunity, is timely in view of recently formulated NIH policy requiring sex balancing in animal studies [19]. Such balancing will cause increased complexity in vaccine design and may require study of the microbiome and in-depth examination of immune responses beyond mere quantitation of functional activities. This approach may provide better understanding of vaccine protective mechanisms. The knowledge gained can be applied to future sex-balanced pre-clinical studies and clinical vaccine trials, critically important as women harbor ~50% of HIV infections worldwide [69].

## Materials and Methods

### Ethics statement

All animal experiments were approved by Institutional Animal Care and Use Committees prior to study initiation. During the course of this study, the study animals were housed in three facilities, each of which approved the work (Bioqual, Inc., Rockville, MD, Protocol No. 12-3507-15; Advanced BioScience Laboratories, Inc. (ABL), Rockville, MD, Protocol No. AUP526; and the NCI Animal Facility, Bethesda, MD, Protocol No. VB007). Each of these facilities is accredited by the Association for Assessment and Accreditation of Laboratory Animal Care International. The standard practices closely follow recommendations made in the Guide for the Care and Use of Laboratory Animals of the United States—National Institutes of Health. The rhesus macaques (*Macaca mulatta*) used in this study were housed in accordance with the recommendations of the AAALAC Standards and with the recommendations in the Guide for the Care and Use of Laboratory Animals. When immobilization was necessary, the animals were anesthetized with approximately 10 mg/kg of ketamine hydrochloride injected intramuscularly. All efforts were made to minimize discomfort of all animals used in the study, including provision of peri-operative and post-operative analgesia and strict accordance to humane endpoint criteria. Details of animal welfare and steps taken to ameliorate suffering were in accordance with the Guide and the recommendations of the Weatherall report, ‘‘The use of non-human primates in research”, as approved by the relevant IACUCs. Animals were housed in temperature controlled facilities with an ambient temperature of 21–26°C, a relative humidity of 30%– 70% and a 12 h light/dark cycle. Due to the nature of the experiment the animals were housed singly in stainless steel wire-bottomed cages and provided with a commercial primate diet and fresh fruit twice daily, with water freely available at all times. All animals were monitored twice daily for activity, food and water intake, and overall health. Enrichment in the form of rotating toys, visual and auditory stimuli, and foraging opportunities were provided daily. Animals that reached IACUC defined endpoints, including pain or distress, that could not be alleviated therapeutically, were humanely euthanized with an overdose of barbiturate consistent with the recommendations of the most recent American Veterinary Medical Association Panel on Euthanasia.

### Animals, immunization and challenge

Sixty Indian rhesus macaques (*Macaca mulatta*) aged 2 to 7 years and negative for SIV, SRV, and STLV were used in this study. Males and females (see below) were assigned to immunization and control groups to achieve similar mean ages, and balanced for Mamu A*01 and B*08 haplotypes (3 Mamu A*01, 2 Mamu B*08, and 1MamuA*01/B*08). Experimental and control groups (Fig 1A) were divided in two for the vaccination phase of the study, and macaques were housed and handled at either Bioqual Inc, or ABL. The challenge of all 60 macaques was conducted at the ABL facility. Post-challenge monitoring after macaques had received up to nine challenges was carried out at the NCI Animal Facility. The number of macaques used was based on a power analysis which determined that using 18 additional historical controls previously challenged with the stock to be used, and the historical infection rate, the estimated power to detect differences between the experimental groups and the controls was 84%. Twenty four macaques were included in each immunization group and primed at weeks 0 (intranasally and orally) and 12 (intratracheally) with three replication-competent Ad5hr recombinants separately encoding SIV_smH4_ *env* (gp140)/*rev*, SIV_239_ *gag* and SIV_239_ *nef*Δ_1–13_ (Fig 1A). The recombinants were administered in PBS at 5 X 10^8^pfu/dose/route as previously described [24]. The SIV_mac239_ monomeric gp120 and oligomeric gp140 boosting immunogens were produced in CHO cells, purified and characterized as previously described [70], and administrated intramuscularly with MF59 adjuvant (Novartis Vaccines and Diagnostics, Cambridge, MA) at weeks 39 and 51. The gp120 immunization group (16 females and 8 males) received 100μg/dose of monomeric SIV_mac239_ gp120 in MF59 adjuvant, and the gp140 immunization group (16 females and 8 males) received 100μg/dose of oligomeric SIV_mac239_ gp140 in MF59. Control macaques (7 females and 5 males) received equivalent doses of Ad5hrΔE3 empty vector and MF59 adjuvant only. At week 59, all macaques were challenged intrarectally using a repeated low dose of SIV_mac251_ (1:500 dilution; 120 TCID_50_), a challenge stock developed by Dr. Ronald Desrosiers and provided by Dr. Nancy Miller, Division of AIDS, NIAID. As SIV exposures were intrarectal, we did not synchronize females prior to initiating challenges. Challenges were continued weekly until the onset of infection determined by a plasma viral load of ≥50 SIV RNA copies/ml as assessed by the NASBA method [71–72]. Macaques were monitored for 40 weeks after infection or until euthanasia criteria were met. Samples from all macaques were included in each analysis except as specified in individual figure legends. Experiments were not blinded. By the time this study was completed, 53 additional historical control rhesus macaques, challenged intrarectally repeatedly with a 1:500 dilution of the same SIV_mac251_ stock, were available to provide greater statistical power for the analyses. Twenty-three of these macaques have been reported in previous publications [73,74]. Data on the remaining 30 have not yet been published. Additionally, rectal pinch biopsies were obtained at necropsy from 6 chronically SIV infected rhesus macaques for use in validating the staining of Env-specific rectal memory B cells.

### Antibody binding assays: binding titers and antibody to SIV_mac251_ cyclic V2 peptide

Serum antibody binding titers to monomeric SIV_mac239_ gp120, oligomeric SIV_mac239_ gp140 (Novartis) and SIV_smH4_ gp120 protein (ABL) were assessed by ELISA as described previously [75]. Antibody titer was defined as the reciprocal of the serum dilution at which the optical density (OD) of the test serum was two times greater than that of the negative-control serum diluted 1:50. Binding antibody end point titers to variable region V2 of SIV gp120 Env were analyzed in serum samples collected prior to immunization and 2 weeks after the second protein boost (wk 53) by ELISA as previously described [7] using a peroxidase-labeled γ chain specific goat anti-monkey IgG (Catalog No 074-11-021, KPL, Gaithersburg, MD) and a custom-synthesized SIV_mac251_ cyclic V2 full-length peptide: CIAQNNCTGLEQEQMISCKFNMTGLKRDKTKEYNETWYSTDLVCEQGNSTDNESRCY (JPT Peptide Technologies, GmbH, Berlin, Germany).

### Neutralizing and non-neutralizing antibody activities

Serum neutralizing antibody titers against SIV_mac251.6_ (tier 1) and SIV_mac251.30_ (tier 3) were assayed in TZM-bl cells as described [76]. Neutralizing titers were defined as the reciprocal serum dilution at which there was a 50% reduction in relative luminescence units compared to virus control wells which contained no test sample.

Serum antibody-dependent cell-mediated cytotoxicity (ADCC) was evaluated using a rapid fluorometric assay [77]. Briefly, CEM-NK^R^ cells coated with SIV_mac239_ gp120 or SIV_mac239_ gp140 (Novartis) were used as targets along with human effector PBMC at an effector-to-target (E:T) ratio of 50:1, and serially diluted macaque sera. Controls included unstained and single-stained target cells. The percent ADCC cell killing was determined by back-gating on the PKH-26^high^ population of targets cells that lost the CFSE viability dye. ADCC titers are defined as the reciprocal dilution at which the percent ADCC killing was greater than the mean percent killing of the negative controls plus three standard deviations. The maximum % killing for each serum was determined. Results were expressed as the 50% maximum killing titer: the reciprocal serum dilution at which 50% maximum killing was observed, and as endpoint titers.

Antibody-dependent cellular phagocytosis (ADCP) activity was measured as previously described [78], with minor modifications. Briefly, SIV_mac239_ gp120 or SIV_mac239_ gp140 was biotinylated with the Biotin-XX Microscale Protein Labeling Kit (Life Technologies, Grand Island, NY), and 3–5 μg of gp120 or gp140 was incubated with a 100-fold dilution of 1μm Yellow-Green streptavidin-fluorescent beads (Life Technologies) for 25 min at room temperature in the dark. Serial dilutions of each serum sample (1:50 to 1:3000) were added to 250,000–300,000 THP-1 cells in wells of a 96-well U-bottom plate. The bead-gp120/gp140 mixture was further diluted 5-fold in RPMI 1640 medium containing 10% fetal bovine serum (R10) and 50 μl was added to the cell/serum mixtures and incubated for 3 h at 37°C. Cells were then washed at low speed, fixed in 2% PFA, and assayed for fluorescent bead uptake by flow cytometry using a BD Biosciences LSRII. The phagocytic score of each sample was calculated as follows: (% phagocytosis x MFI)/10^6^. The values were standardized to background values (cells and bead only without serum) by dividing the phagocytic score of the test sample by the phagocytic score of the background sample.

### SIV-specific IgA and IgG antibodies in rectal secretions

Rectal secretions were collected using cotton swabs and stored in 1 ml of PBS containing 0.1% bovine serum albumin, 0.01% thimerosal, and 750 Kallikrein inhibitor units of aprotinin at -70°C until analyzed. Samples were tested for blood contamination using Chemstrips 5 (Boehringer Mannheim) prior to assay. To remove fecal contaminant sample was passed through a 5μm PVDF microcentrifugal filter unit (Millipore, Billerica, MA). Briefly, SIVgp120 and gp140-specific IgA and IgG antibodies were measured by ELISA as previously described [79,80]. Env-specific IgA and IgG standards derived from IgG-depleted pooled serum or purified serum IgG, respectively, obtained from SIV_mac251_-infected macaques and quantified as previously described [27] were used to generate standard curves. HRP-conjugated goat anti-monkey IgA and IgG (Nordic Immunology) and TMB substrate were used in sequential steps, followed by the addition of phosphoric acid prior to reading the OD at 450 nm. Total IgA and IgG antibodies were measured in each sample and used to standardize gp120 or gp140-specific IgA and IgG concentrations. Results are reported as Env-specific IgA or IgG/total IgG or IgA (ng specific/μg total).

### SIVgp120 and gp140-specific rectal B cells

Rectal biopsies were collected at different time points and single cell suspensions were obtained from fresh samples as previously described [21]. Cells obtained were stained with a mixture of fluorescent-conjugated monoclonal antibodies. Env-specific memory B cells were identified using a biotinylated SIV_mac239_ gp120 or gp140 with the Biotin-XX Microscale Protein Labeling Kit (Life Technologies, Grand Island, NY) followed by APC-conjugated Streptavidin (Life Technologies) as previously described [20]. Briefly, staining was carried out at 4°C in the presence of unconjugated anti-CD4 antibodies to block reactivity to CD4. Representative gating is illustrated in S14A Fig. gp120/gp140-specific B cells were detected within the memory B cell subpopulation (CD27^+/-^IgD^-^). Rectal plasmablasts and plasma cells were similarly assessed in fresh rectal biopsies as previously described [21]. Plasmablasts were identified as CD19^+^CD20^+/-^IgD^-^IRF4^+^CD138^-^HLA-DR^+^Ki67^+^ and plasma cells as CD19^+^CD20^+/-^IgD^-^ IRF4^+^CD138^+^HLA-DR^-^Ki67^-^ (S14B Fig). The Env-specific memory B cell staining was validated using rectal pinch biopsies from 6 chronically SIV infected rhesus macaques (not a part of this study) in analyses by both flow cytometry and B cell ELISPOT. A significant correlation was obtained (S14C Fig).

### SIVgp120 and gp140-specific antibody secreting bone marrow cells

Bone marrow samples were collected at different time points, and lymphocytes were purified as previously described [75] and frozen until analysis. Lymphocytes were thawed and both total and SIVgp120 or gp140-specific IgG and IgA secreting B cells were quantified by ELISpot as previously described [37]. Briefly, plasmablasts and plasma cells were quantified on unstimulated samples while memory B cells were enumerated following 3 days of polyclonal stimulation with CpG (ODN-2006) (Operon), 0.5 μg/ml recombinant human sCD40L (Peprotech), and 50 ng/ml recombinant human IL-21 (Peprotech). In both cases, Env-specific IgA and IgG antibody secreting cells (ASC) were standardized to the total number of IgA and IgG ASC and are reported as the percentage of SIVgp120 or gp140-specific ASC relative to the number of total ASC.

### Intracellular cytokine assay

Peripheral blood mononuclear cells (PBMC) were isolated from EDTA-treated blood by ficoll gradient [79] and frozen until assay. Cellular immune responses were evaluated by intracellular staining for SIV-specific IFN-γ, IL-2 and TNF-α cytokine secreting cells. After thawing, PBMC were stimulated with peptides at 1μg/ml final concentration. SIV peptide pools were made up of 15 mers overlapping by 11 amino acids and included Env_smH4_ (Advanced BioScience Laboratories, Inc), Env_mac239_, Gag_mac239_ (AIDS Research Reference and Reagents Program) and Nef_mac251_. Control tubes included a non-stimulated and a Leucocyte activation Cocktail (BD Pharmingen) as a positive control. Anti-CD28 PE/Texas red (clone CD28.2; Beckman Coulter) and anti-CD49d (clone 9F10; eBioscience) were also added during stimulation along with a protein transport inhibitor (BD Pharmingen). After 6h incubation at 37°C, cells were washed with PBS, then stained as previously described [33] with the following antibodies: Anti-CD4 PerCP/Cy5.5 (clone L220), Anti-CD8 Qdot655 (clone RPA-T8, eBioscience), and Anti-CD95 PE/Cy5 (clone DX2, eBioscience). A viability dye (Life Technologies) was added to the antibody cocktail to exclude dead cell background. Following incubation for 30 min at 4°C in the dark, intracellular staining was performed. Cells were washed twice, resuspended in 250μl fix/perm solution (BD Pharmingen) for 20 min at 4°C, washed twice with BD perm/wash buffer and resuspended in 100μl wash buffer plus the following antibodies: Anti-CD3 Pacific blue (SP34-2, BD Pharmingen), Anti- IFN-γ APC (B27, BD Pharmingen), Anti-TNF-α FITC (Mab11, BD Pharmingen) and Anti-IL-2 PE (MQ1-17H12, BD Pharmingen). After 30 min at 4°C in the dark, cells were washed twice with BD perm/wash buffer and pellets were resuspended in 2% formaldehyde solution for acquisition on an LSRII. CD3^+^ T cells were used as a gate for CD4^+^ and CD8^+^ T cells, and each population was further divided into CD28^+^CD95^+^ central memory (CM) and CD28^-^CD95^+^ effector memory (EM) cells. The percent of cytokine-secreting cells in each memory cell subset was determined following subtraction of the values obtained with non-stimulated samples. Both subsets were summed to give the total memory (TM) T-cell population. Flow-cytometric analysis was performed using FlowJo V9.8.1. (ThreeStar, Ashland, OR).

### Statistical analyses

All tests of quantitative data are rank-based and thus distribution-free, so the weak assumptions of the tests are met. Rank-based tests do not require similar variances. Grouped, continuous, and discrete data were analyzed using methods appropriate to each of those types. The Wilcoxon rank-sum analysis was used to test for differences between immunization groups for binding antibody titers, neutralizing and non neutralizing antibody activities, rectal SIV-Env specific B cells, mucosal Env-specific IgG and IgA, Env-specific antibody secreting B cells and cytokine responses. The Wilcoxon signed-rank test was used to test for differences in paired samples within immunization groups. The Cochran-Armitage test was used to analyze V2 peptide titers and ADCC titers. The Spearman rank correlation test was used to assess the relationships of antibody and cellular responses with number of challenges and viral loads. Acquisition and survival data were analyzed using the exact logrank test. For all comparisons a two-sided p<0.05 was considered statistically significant. Adjustments for multiple comparisons were not made. Estimates of variation are provided as needed in individual figure legends. Analyses were conducted using SAS/STAT software version 9.3 and GraphPadPrism V6.

## Supporting Information

S1 Fig Comparison of rates of SIV acquisition in immunized male and female macaques. (A) Delayed SIV acquisition in all immunized females compared to immunized males. (B) Delayed SIV acquisition in gp120-immunized females compared to gp120-immunized males but (C) not in gp140-immunized females compared to gp140-immunized males.
(PDF)

S2 Fig Serum Env-specific binding antibody responses in immunized macaques. Binding antibody titers in gp120- (A) and gp140-immunized (B) macaques to SIV_mac239_ monomeric gp120 and oligomeric gp140 and comparison of gp140 titers (C) between immunization groups and controls over the course of immunization and 2wkpi. Binding titers of pre-bleed samples were not obtained, but titers of control samples at all time points were <50. (D) Binding antibody titers to cyclic V2 peptide prior to immunization and at wk 53 by immunization group.
(PDF)

S3 Fig Serum neutralizing and non-neutralizing antibody activities. (A) Neutralizing antibody titers over the course of immunization and 2wkpi by immunization group. ADCC to gp120 and gp140 targets expressed as 50% maximum killing titer (B) and endpoint titer (C) at wk 53. Mean phagocytosis score/background phagocytosis to gp140 targets at wk 53 and 2wkpi (D) and to gp120 targets at wk 53 (E) by immunization group. * *p = 0*.*0034*, **p <0.*0001*. All titers expressed as geometric mean with 95% CL; phagocytosis expressed as mean ± SEM.
(PDF)

S4 Fig Rectal Env-specific IgA and IgG 2wk post 2^nd^ priming (wk 14) and 2 wk post 2^nd^ boost (wk 53). (A) IgA and (B) IgG reactivity to gp120 and gp140 at wk 14 and IgA (C)and IgG (D) reactivity to gp120 and gp140 at wk 53 by immunization group. All results were expressed as ng specific Ig/μg total Ig and then standardized to control levels. Mean values ± SEM are shown.
(PDF)

S5 Fig Bone marrow Env-specific memory ASC assessed by ELISpot. Memory B cells secreting Env-specific IgG (A) and IgA (B) over the immunization course and 2wkpi. Comparison of Env-specific IgG and IgA memory B cells for both immunization groups at wk 53 (C) and 2wkpi (D). The gp120 group was tested against monomeric gp120 and the gp140 group against oligomeric gp140. *p<0.01. Due to the large number of tests performed, only differences with p values <0.01 are shown in panels A and B. Mean values ± SEM are shown.
(PDF)

S6 Fig Bone marrow Env-specific plasmablasts/plasma cells ASC assessed by ELISpot. PB/PC secreting Env-specific IgG (A) and IgA (B) over the immunization course and 2wkpi. Comparison of Env-specific IgG and IgA PB/PC for both immunization groups at wk 53 (C) and 2wkpi (D). The gp120 group was tested against monomeric gp120 and the gp140 group against oligomeric gp140. *p < 0.01. Due to the large number of tests performed, only differences with p values < 0.01 are shown in panels A and B. Mean values ± SEM are shown.
(PDF)

S7 Fig Vaccine-induced SIV-specific cellular immune responses by immunization group. PBMC obtained 2wkpi from all macaque groups were assessed by intracellular cytokine staining for Env_smH4_- (A and C) and Env_239_- (B and D) specific CD4^+^ (A,B) and CD8^+^ (C, D) T cells. The frequency of CD4^+^ (E and F) and CD8^+^ (G and H) T cells specific for immunizing antigens in the Ad-recombinants (Env_smH4_, Gag_239_ and Nef_239_) or protein boosts (Env_239_) were summed and presented. CD4^+^ and CD8^+^ central and effector memory cells were summed and results for total memory (TM) CD4^+^ or CD8^+^ T cells are presented as the percent cytokine positive cells expressing IFN-γ, TNF-α, and/or IL-2. Due to the large number of macaques, half the macaques in each group were assessed for either SIV_smH4_ or SIV_mac239_ Env-specificity and half were assessed for either SIV_mac239_ Gag or SIV_mac251_ Nef specificity. Mean values + SEM are shown.
(PDF)

S8 Fig Systemic neutralizing and cyclic V2 binding antibody titers by sex. Neutralizing antibody titers in (A) gp120- and (B) gp140-immunized females and males. (C) Serum cyclic V2 binding antibody titers in females and males of all macaque groups at 2 weeks post 2nd boost (wk 53). Bars denote geometric means with 95% CL.
(PDF)

S9 Fig Bone marrow Env-specific plasmablasts/plasma cells and memory B cells induced by vaccination in females and males. Env-specific plasmablasts/plasma cells and memory B cells secreting Env-specific IgG (A,C) and IgA (B,D) in gp120- (A,B) and gp140- (C,D) immunization groups by sex are shown. The gp120 group was tested against monomeric gp120 and gp140 group against oligomeric gp140 by ELISpot. Closed black symbols represent females and open red symbols represent males. Mean values ± SEM are shown.
(PDF)

S10 Fig Env-specific IgA and IgG in rectal secretions of vaccinated females and males. Env-specific IgA (A, C) and IgG (B, D) for gp120- (A, B) and gp140- (C, D) immunized females and males are shown over the course of immunization and 2wkpi. The gp120 group was tested against monomeric gp120 and the gp140 group against oligomeric gp140. All results were standardized to background control levels. Bars represent mean values ± SEM.
(PDF)

S11 Fig SIV-specific cellular immune responses in females and males. PBMC obtained 2wkpi from all macaque groups were assessed by intracellular cytokine staining for Env_smH4_- (A,C) and Env_239_- (B,D) specific CD4^+^ (A,B) and CD8^+^ (C,D) T cells. Results for all females and all males are shown. Additionally, the frequency of CD4^+^ (E,F) and CD8^+^ (G,H) T cells specific for immunizing antigens in the Ad-recombinants (Env_smH4_, Gag_239_ and Nef_239_) or protein boosts (Env_239_) were summed and presented for all females and all males. CD4^+^ and CD8^+^ central and effector memory cells were summed and results for total memory(TM) CD4^+^ or CD8^+^ T cells are presented as the percent cytokine positive cells expressing IFN-γ, TNF-α and/or IL-2. Due to the large number of macaques, half the macaques in each group were assessed for either SIV_smH4_ or SIV_mac239_ Env-specificity and half were assessed for either SIV_mac239_ Gag or SIV_mac251_ Nef specificity. Mean values + SEM are shown.
(PDF)

S12 Fig Rectal Env-specific IgG not correlated with delayed SIV acquisition in immunized macaques. No influence of rectal Env-specific IgG at wk 55 on the rate of acquisition in (A) all immunized macaques, (B) gp120-immunized or (C) gp140-immunized macaques, (D) all immunized females, (E) gp120- immunized or (F) gp140- immunized females, (G) all immunized males, (H) gp120-immunized or (I) gp140-immunized males.
(PDF)

S13 Fig Rectal Env-specific IgA not correlated with delayed SIV acquisition in immunized males. No influence of rectal Env-specific IgA at wk 55 on the rate of infection in (A) all immunized males, (B) gp120-immunized males, and (C) gp140- immunized males.
(PDF)

S14 Fig Gating strategy for Env-specific memory B cells, plasmablasts and plasma cells in rectal tissue. (A) Example of flow cytometry staining for Env-specific memory B cells: Live CD2^-^CD14^-^ cells from rectal pinches were gated for CD19^+^CD20^+^ B cells and then IgD^+^ B cells were excluded. The far right-hand plot shows Env-specific memory B cells in a vaccinated macaque and a control macaque using biotinylated gp120. (B) Example of flow cytometry staining for PB and PC. Live CD2^-^CD14^-^ cells from rectal pinches were gated for CD19^+^CD20^+^ B cells and then IgD^+^ B cells were excluded. IgD^-^ B cells were further gated for IRF4^+^CD138^-^ and IRF4^+^ CD138^+^. PB (upper-right quadrant highlighted by the red box) are identified as CD19^+^ CD20^+/-^IgD^-^IRF4^+^ CD138^-^ HLA-DR^+^Ki67^+^. PC (lower left quadrant highlighted by the red box) are identified as CD19^+^ CD20^+/-^IgD^-^IRF4^+^CD138^+^HLA-DR^-^Ki67^-^. (C) gp120-specific memory B cells quantified by flow cytometry correlate with frequency of Env-specific memory B cells secreting IgG + IgA by ELISpot in rectal biopsies from chronically SIV infected macaques.
(PDF)

S15 Fig Individual plasma viral loads for gp120 and gp140 immunized and control macaques. (A-C) Viral loads were recorded up to 40 wkpi. Female macaques are shown in black lines and males in red lines. A † marks macaques that were euthanized before 40 weeks of follow up. Macaque R663 in the gp140-immunized group resisted infection over 9 challenges, and is not shown.
(PDF)

S16 Fig Dynamics of plasma viral loads in SIV-infected historical and current control macaques. Plasma viral loads (geometric mean) in historical and current controls (A) and in (B) combined control males and females.
(PDF)

S17 Fig No correlation of serum neutralizing and ADCC antibody activities with viremia control. Lack of correlation of serum (wk 53) ADCC against gp120 and gp140 targets expressed as 50% maximum killing titer from (A and B) gp120- and (C and D) gp140-immunized animals with peak viral load. No correlation of neutralizing antibody titers (wk 57) from (E) gp120- and (F) gp140-immunized animals with peak viral load.
(PDF)

S18 Fig SIV Env-specific CD8^+^ T cell responses correlate with control of viremia in all males, but not in females. PBMC obtained 2wkpi from all macaque groups were assessed by intracellular cytokine staining for Env_smH4_-specific CD8^+^ T cells. Significant correlations were observed in all males with control of viremia (A) including reduced peak, median acute (weeks 1–6) and median chronic (over wk 8–24) viral loads. Similar correlations were not seen for all female macaques (B). CD8^+^ central and effector memory cells were summed and results for total memory (TM) CD8^+^ T cells are presented as the percent cytokine positive cells expressing IFN-γ, TNF-α and / or IL-2. Due to the large number of macaques, only half the macaques in each group were assessed for SIV_smH4_ Env-specificity.
(PDF)

S19 Fig Dynamics of plasma viral loads and CD4 counts in SIV- infected female and male rhesus macaques by immunization group. Plasma viral loads (geometric mean) in females (A) and males (B) by immunization group. Absolute CD4^+^ T cell counts (mean values) in (C) females and (D) males by immunization group. *p < 0.05 for immunized males vs controls; p < 0.01 for immunized females vs controls.
(PDF)
