# Supplementary material for: Mucosal B Cells Are Associated with Delayed SIV Acquisition in Vaccinated Female but Not Male Rhesus Macaques Following SIVmac251 Rectal Challenge
Source: PLoS Pathog. 2015 Aug 12;11(8):e1005101. doi: 10.1371/journal.ppat.1005101 (PMC4534401; doi:10.1371/journal.ppat.1005101)
Supplement: S1 Fig — (A) Delayed SIV acquisition in all immunized females compared to immunized males. (B) Delayed SIV acquisition in gp120-immunized females compared to gp120-immunized males but (C) not in gp140-immunized females compared to gp140-immunized males. (PDF) [file ppat.1005101.s001.pdf]

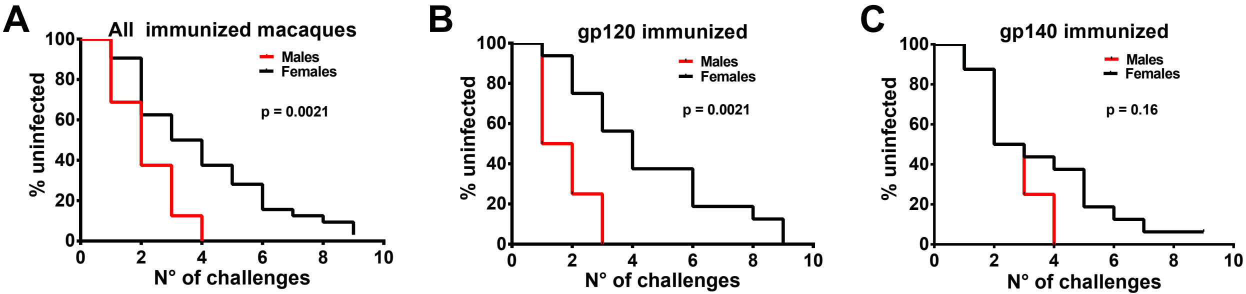

S1 Fig. Comparison of rates of SIV acquisition in immunized male and female macaques. (A) Delayed SIV acquisition in all immunized females compared to immunized males. (B) Delayed SIV acquisition in gp120-immunized females compared to gp120-immunized males but (C) not in gp140-immunized females compared to gp140-immunized males.
