# Supplementary material for: Mucosal B Cells Are Associated with Delayed SIV Acquisition in Vaccinated Female but Not Male Rhesus Macaques Following SIVmac251 Rectal Challenge
Source: PLoS Pathog. 2015 Aug 12;11(8):e1005101. doi: 10.1371/journal.ppat.1005101 (PMC4534401; doi:10.1371/journal.ppat.1005101)
Supplement: S2 Fig — Binding antibody titers in gp120- (A) and gp140-immunized (B) macaques to SIVmac239 monomeric gp120 and oligomeric gp140 and comparison of gp140 titers (C) between immunization groups and controls over the course of immunization and 2wkpi. Binding titers of pre-bleed samples were not obtained, but titers of control samples at all time points were <50. (D) Binding antibody titers to cyclic V2 peptide prior to immunization and at wk 53 by immunization group. (PDF) [file ppat.1005101.s002.pdf]

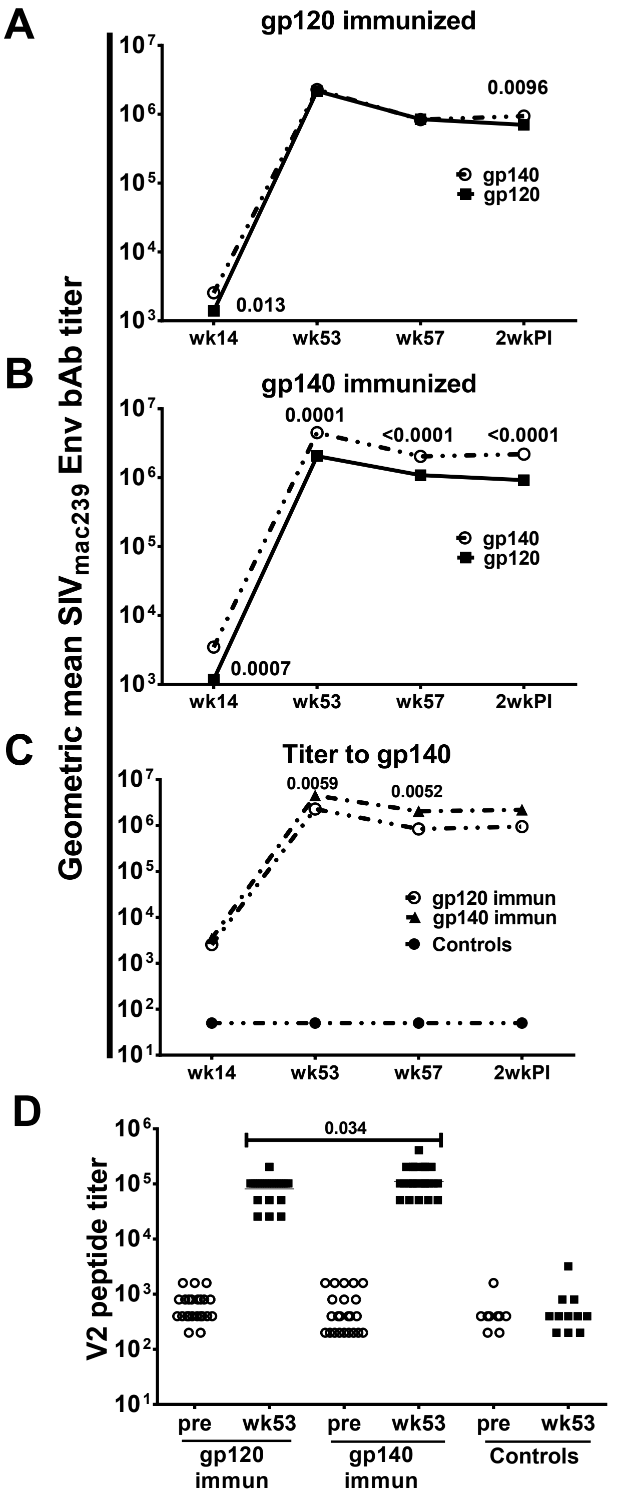

S2 Fig. Serum Env-specific binding antibody responses in immunized macaques. Binding antibody titers in gp120 - (A) and gp140 - immunized (B) macaques to SIV_{mac239} monomeric gp120 and oligomeric gp140 and comparison of gp140 titers (C) between immunization groups and controls over the course of immunization and 2wkpi. Binding titers of pre -bleed samples were not obtained, but titers of control samples at all time points were <50. (D) Binding antibody titers to cyclic V2 peptide prior to immunization and at wk 53 by immunization group.
