# Supplementary material for: Mucosal B Cells Are Associated with Delayed SIV Acquisition in Vaccinated Female but Not Male Rhesus Macaques Following SIVmac251 Rectal Challenge
Source: PLoS Pathog. 2015 Aug 12;11(8):e1005101. doi: 10.1371/journal.ppat.1005101 (PMC4534401; doi:10.1371/journal.ppat.1005101)
Supplement: S3 Fig — (A) Neutralizing antibody titers over the course of immunization and 2wkpi by immunization group. ADCC to gp120 and gp140 targets expressed as 50% maximum killing titer (B) and endpoint titer (C) at wk 53. Mean phagocytosis score/background phagocytosis to gp140 targets at wk 53 and 2wkpi (D) and to gp120 targets at wk 53 (E) by immunization group. * p = 0.0034, **p <0.0001. All titers expressed as geometric mean with 95% CL; phagocytosis expressed as mean ± SEM. (PDF) [file ppat.1005101.s003.pdf]

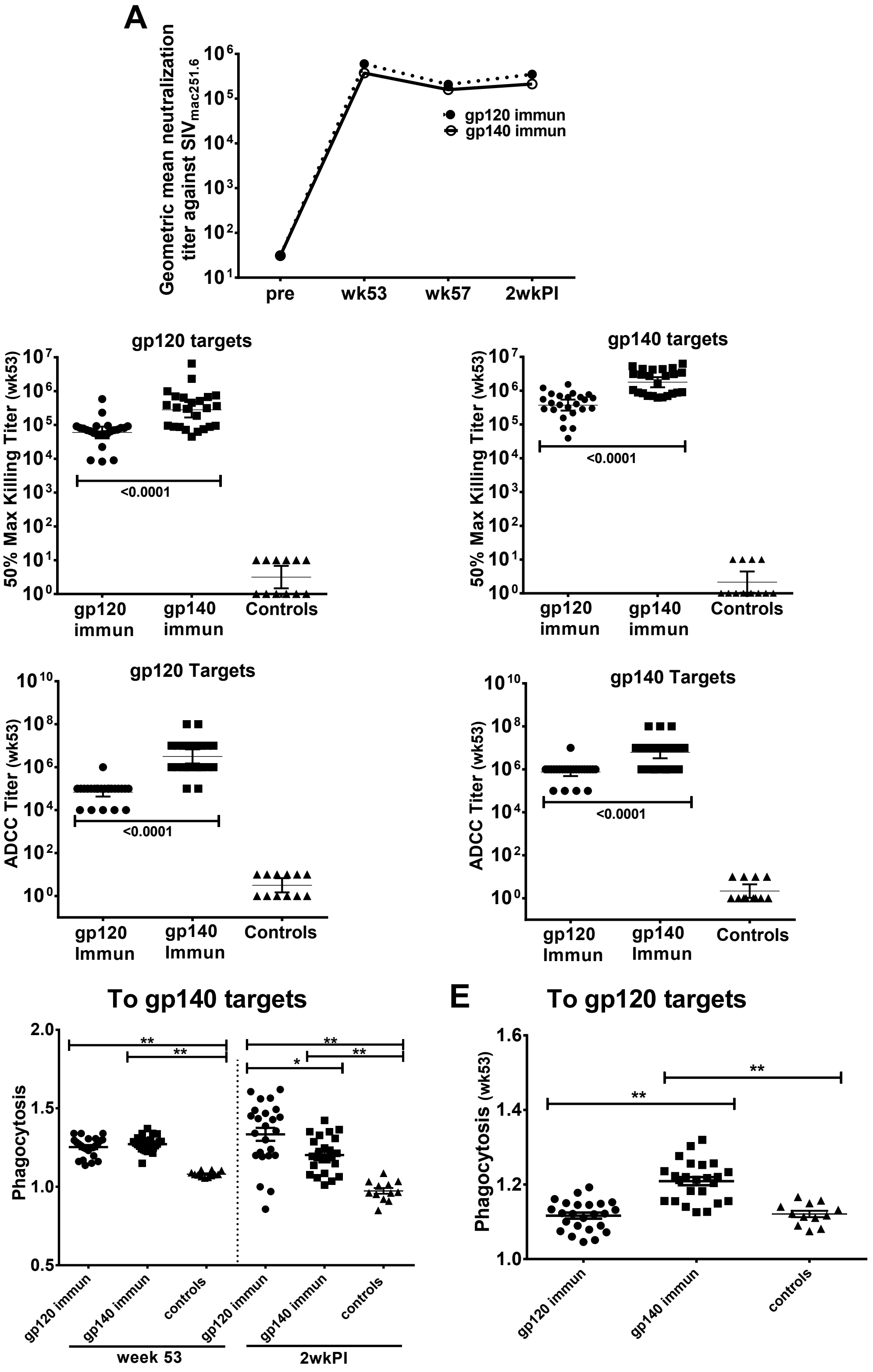

S3 Fig. Serum neutralizing and non- neutralizing antibody activities. (A) Neutralizing antibody titers over the course of immunization and 2wkpi by immunization group. ADCC to gp120 and gp140 targets expressed as 50% maximum killing titer (B) and endpoint titer (C) at wk 53. Mean phagocytosis score / background phagocytosis to gp140 targets at wk 53 and 2wkpi (D) and to gp120 targets at wk 53 (E) by immunization group. * *p = 0.0034*, ***p <0.0001*. All titers expressed as geometric mean with 95% CL; phagocytosis expressed as mean ± SEM.
