# Supplementary material for: Mucosal B Cells Are Associated with Delayed SIV Acquisition in Vaccinated Female but Not Male Rhesus Macaques Following SIVmac251 Rectal Challenge
Source: PLoS Pathog. 2015 Aug 12;11(8):e1005101. doi: 10.1371/journal.ppat.1005101 (PMC4534401; doi:10.1371/journal.ppat.1005101)
Supplement: S4 Fig — (A) IgA and (B) IgG reactivity to gp120 and gp140 at wk 14 and IgA (C)and IgG (D) reactivity to gp120 and gp140 at wk 53 by immunization group. All results were expressed as ng specific Ig/μg total Ig and then standardized to control levels. Mean values ± SEM are shown. (PDF) [file ppat.1005101.s004.pdf]

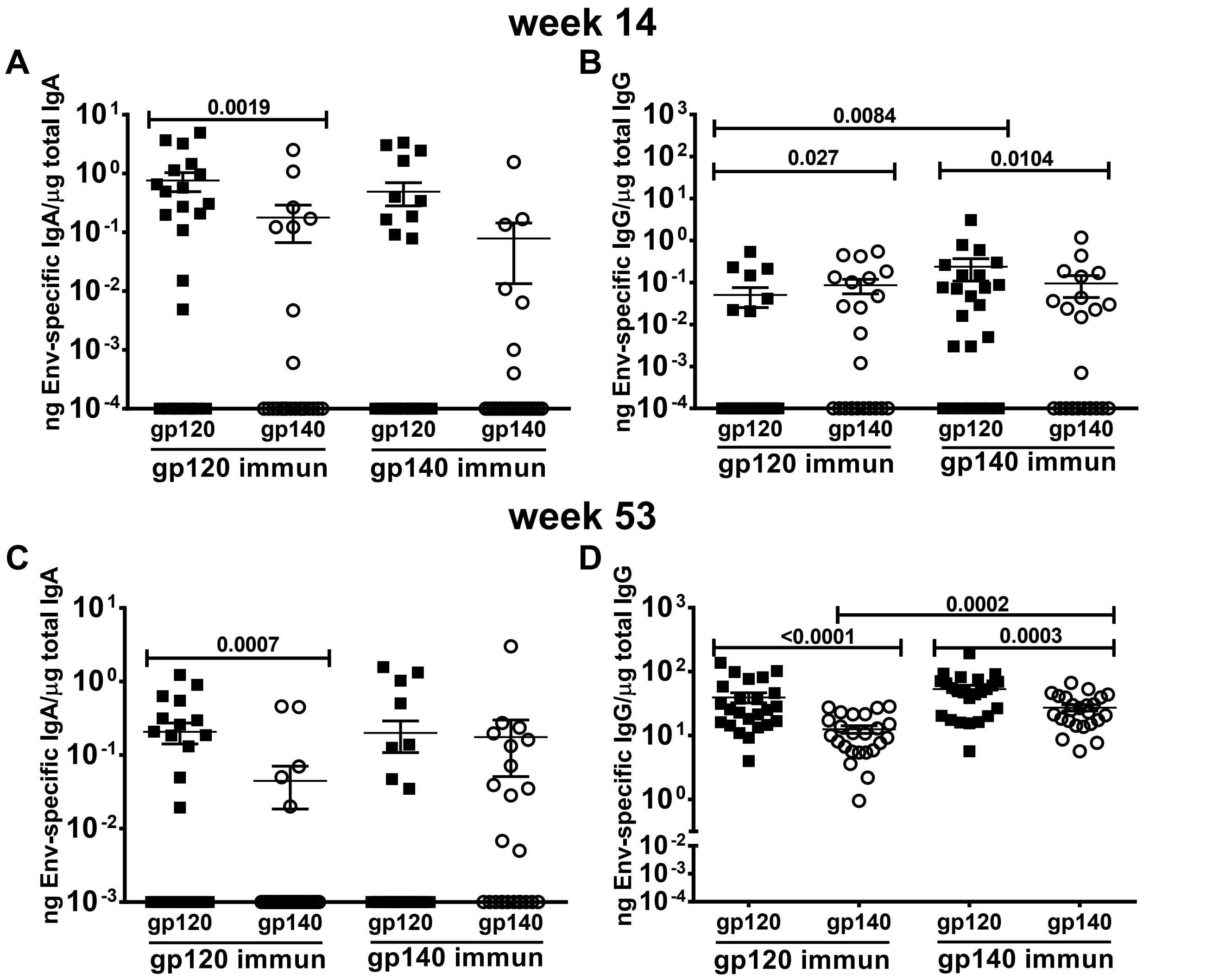

S4 Fig. Rectal Env-specific IgA and IgG 2wk post 2nd priming (wk 14) and 2wk post 2nd boost (wk 53). (A) IgA and (B) IgG reactivity to gp120 and gp140 at wk 14 and IgA (C) and IgG (D) reactivity to gp120 and gp140 at wk 53 by immunization group. All results were expressed as ng specific Ig/μg total Ig and then standardized to control levels. Mean values ± SEM are shown.
