# Supplementary material for: Mucosal B Cells Are Associated with Delayed SIV Acquisition in Vaccinated Female but Not Male Rhesus Macaques Following SIVmac251 Rectal Challenge
Source: PLoS Pathog. 2015 Aug 12;11(8):e1005101. doi: 10.1371/journal.ppat.1005101 (PMC4534401; doi:10.1371/journal.ppat.1005101)
Supplement: S6 Fig — PB/PC secreting Env-specific IgG (A) and IgA (B) over the immunization course and 2wkpi. Comparison of Env-specific IgG and IgA PB/PC for both immunization groups at wk 53 (C) and 2wkpi (D). The gp120 group was tested against monomeric gp120 and the gp140 group against oligomeric gp140. *p < 0.01. Due to the large number of tests performed, only differences with p values < 0.01 are shown in panels A and B. Mean values ± SEM are shown. (PDF) [file ppat.1005101.s006.pdf]

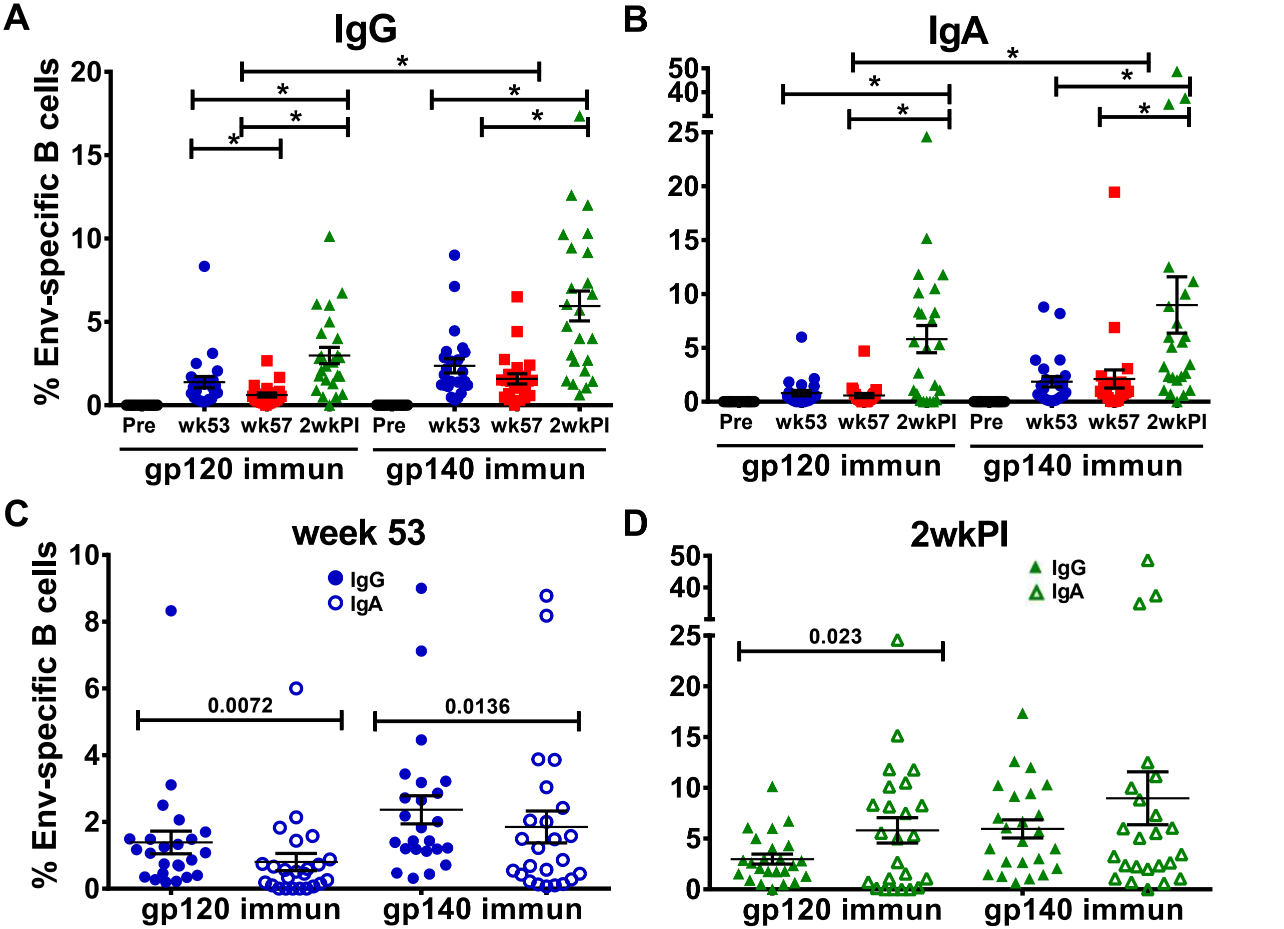

S6 Fig. Bone marrow Env-specific antibody-secreting PB/PC assessed by ELISpot. PB/PC secreting Env-specific IgG (A) and IgA (B) over the immunization course and 2wkpi. Comparison of Env-specific IgG and IgA PB/PC for both immunization groups at wk 53 (C) and 2wkpi (D). The gp120 group was tested against monomeric gp120 and the gp140 group against oligomeric gp140. *$p < 0.01$. Due to the large number of tests performed, only differences with p values < 0.01 are shown in panels A and B. Mean values ± SEM are shown.
