# Supplementary material for: Mucosal B Cells Are Associated with Delayed SIV Acquisition in Vaccinated Female but Not Male Rhesus Macaques Following SIVmac251 Rectal Challenge
Source: PLoS Pathog. 2015 Aug 12;11(8):e1005101. doi: 10.1371/journal.ppat.1005101 (PMC4534401; doi:10.1371/journal.ppat.1005101)
Supplement: S7 Fig — PBMC obtained 2wkpi from all macaque groups were assessed by intracellular cytokine staining for EnvsmH4- (A and C) and Env239- (B and D) specific CD4+ (A,B) and CD8+ (C, D) T cells. The frequency of CD4+ (E and F) and CD8+ (G and H) T cells specific for immunizing antigens in the Ad-recombinants (EnvsmH4, Gag239 and Nef239) or protein boosts (Env239) were summed and presented. CD4+ and CD8+ central and effector memory cells were summed and results for total memory (TM) CD4+ or CD8+ T cells are presented as the percent cytokine positive cells expressing IFN-γ, TNF-α, and/or IL-2. Due to the large number of macaques, half the macaques in each group were assessed for either SIVsmH4 or SIVmac239 Env-specificity and half were assessed for either SIVmac239 Gag or SIVmac251 Nef specificity. Mean values + SEM are shown. (PDF) [file ppat.1005101.s007.pdf]

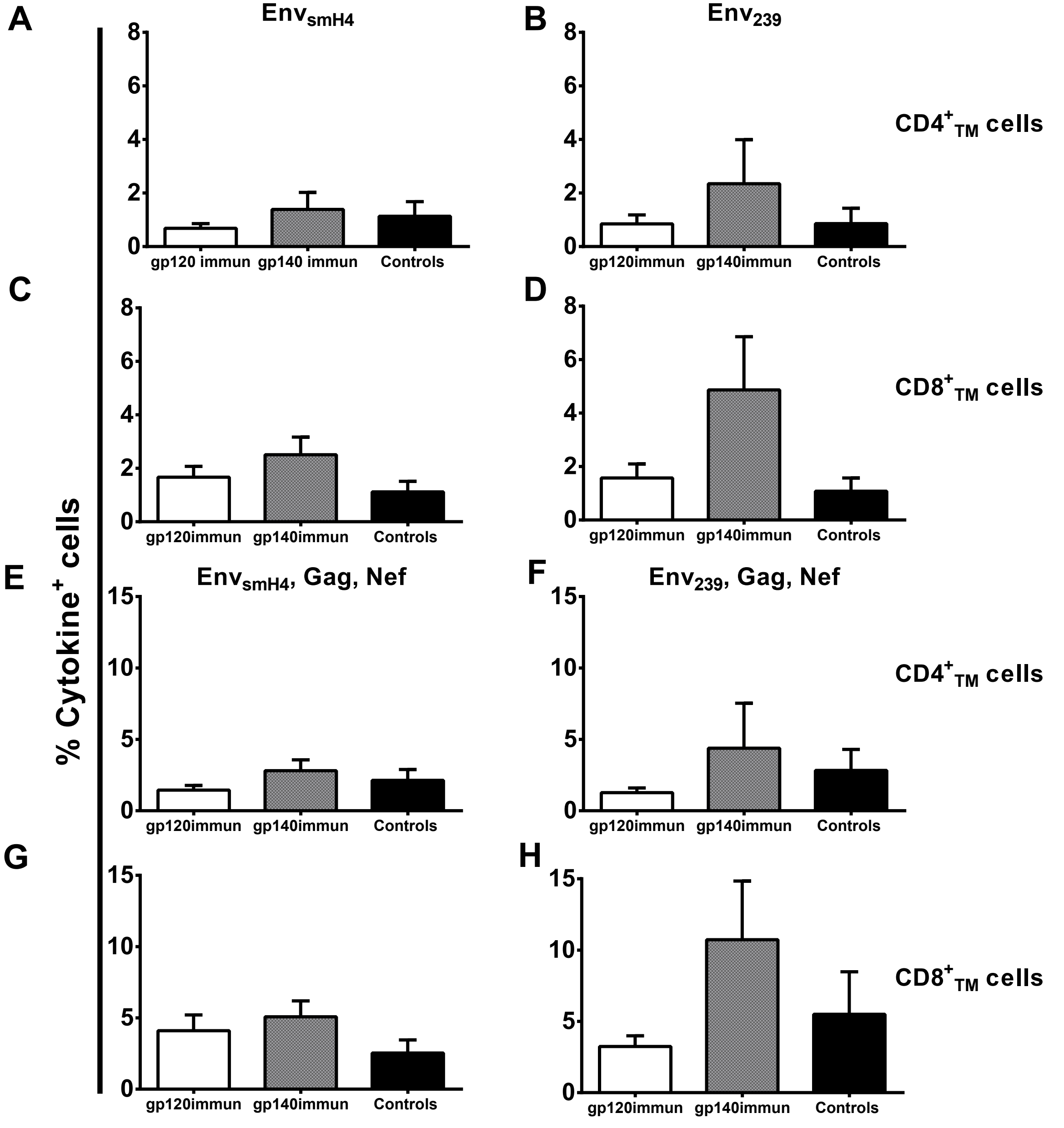

S7 Fig. Vaccine-induced SIV-specific cellular immune responses by immunization group. PBMC obtained 2wkpi from all macaque groups were assessed by intracellular cytokine staining for Env_{smH4}- (A and C) and Env_{239}- (B and D) specific $CD4^+$ (A,B) and $CD8^+$ (C, D) T cells. The frequency of $CD4^+$(E and F) and $CD8^+$ (G and H) T cells specific for immunizing antigens in the Ad - recombinants (Env_{smH4}, Gag_{239} and Nef_{239}) or protein boosts (Env_{239}) were summed and presented. $CD4^+$ and $CD8^+$ central and effector memory cells were summed and results for total memory (TM) $CD4^+$ or $CD8^+$ T cells are presented as the percent cytokine positive cells expressing IFN-γ, TNF-α and/or IL-2. Due to the large number of macaques, half the macaques in each group were assessed for either SIV_{smH4} or SIV_{mac239} Env - specificity and half were assessed for either SIV_{mac239} Gag or SIV_{mac251} Nef specificity. Mean values + SEM are shown.
