# Supplementary material for: Mucosal B Cells Are Associated with Delayed SIV Acquisition in Vaccinated Female but Not Male Rhesus Macaques Following SIVmac251 Rectal Challenge
Source: PLoS Pathog. 2015 Aug 12;11(8):e1005101. doi: 10.1371/journal.ppat.1005101 (PMC4534401; doi:10.1371/journal.ppat.1005101)
Supplement: S8 Fig — Neutralizing antibody titers in (A) gp120- and (B) gp140-immunized females and males. (C) Serum cyclic V2 binding antibody titers in females and males of all macaque groups at 2 weeks post 2nd boost (wk 53). Bars denote geometric means with 95% CL. (PDF) [file ppat.1005101.s008.pdf]

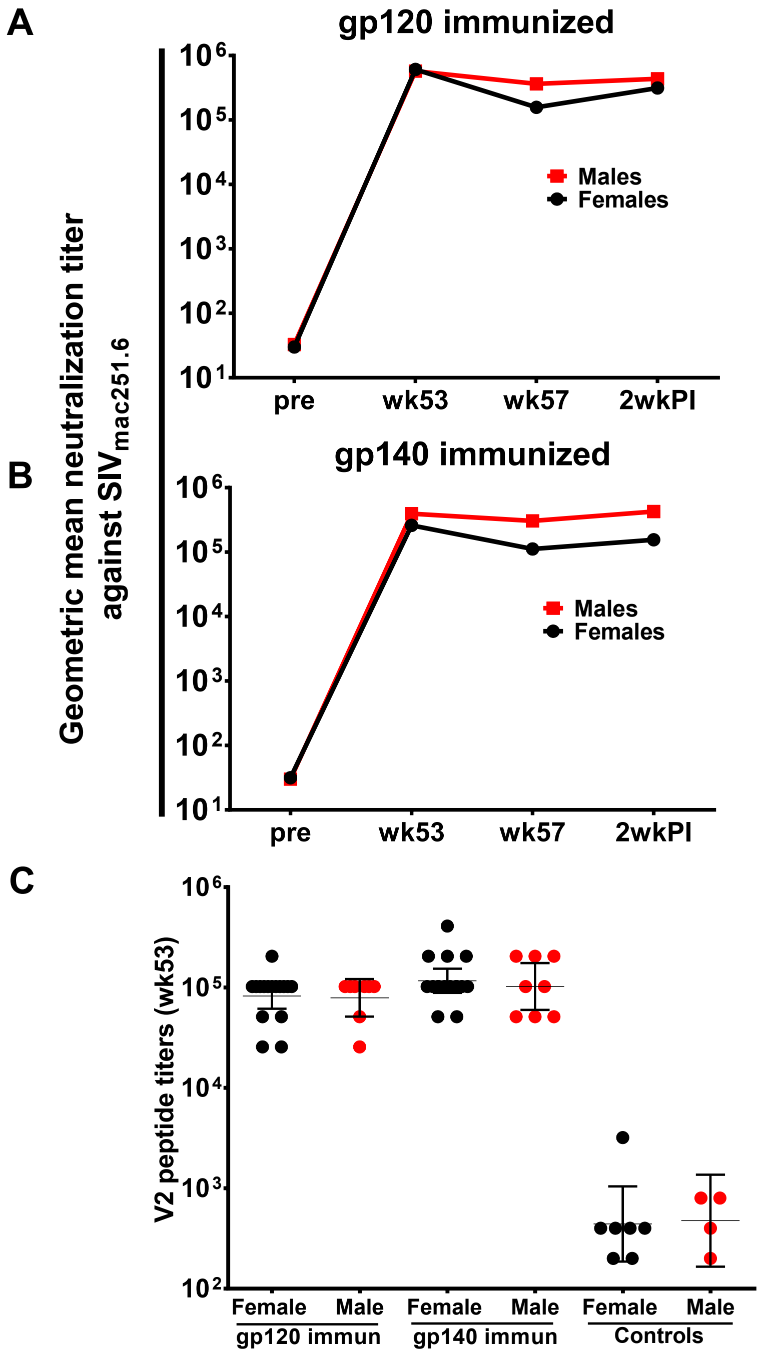

S8 Fig. Systemic neutralizing and cyclic V2 binding antibody titers by sex. Neutralizing antibody titers in (A) gp120 - and (B) gp140 - immunized females and males. (C) Serum cyclic V2 binding antibody titers in females and males of all macaque groups at 2 weeks post 2nd boost (wk 53). Bars denote geometric means with 95% CL.
