# Supplementary material for: Mucosal B Cells Are Associated with Delayed SIV Acquisition in Vaccinated Female but Not Male Rhesus Macaques Following SIVmac251 Rectal Challenge
Source: PLoS Pathog. 2015 Aug 12;11(8):e1005101. doi: 10.1371/journal.ppat.1005101 (PMC4534401; doi:10.1371/journal.ppat.1005101)
Supplement: S9 Fig — Env-specific plasmablasts/plasma cells and memory B cells secreting Env-specific IgG (A,C) and IgA (B,D) in gp120- (A,B) and gp140- (C,D) immunization groups by sex are shown. The gp120 group was tested against monomeric gp120 and gp140 group against oligomeric gp140 by ELISpot. Closed black symbols represent females and open red symbols represent males. Mean values ± SEM are shown. (PDF) [file ppat.1005101.s009.pdf]

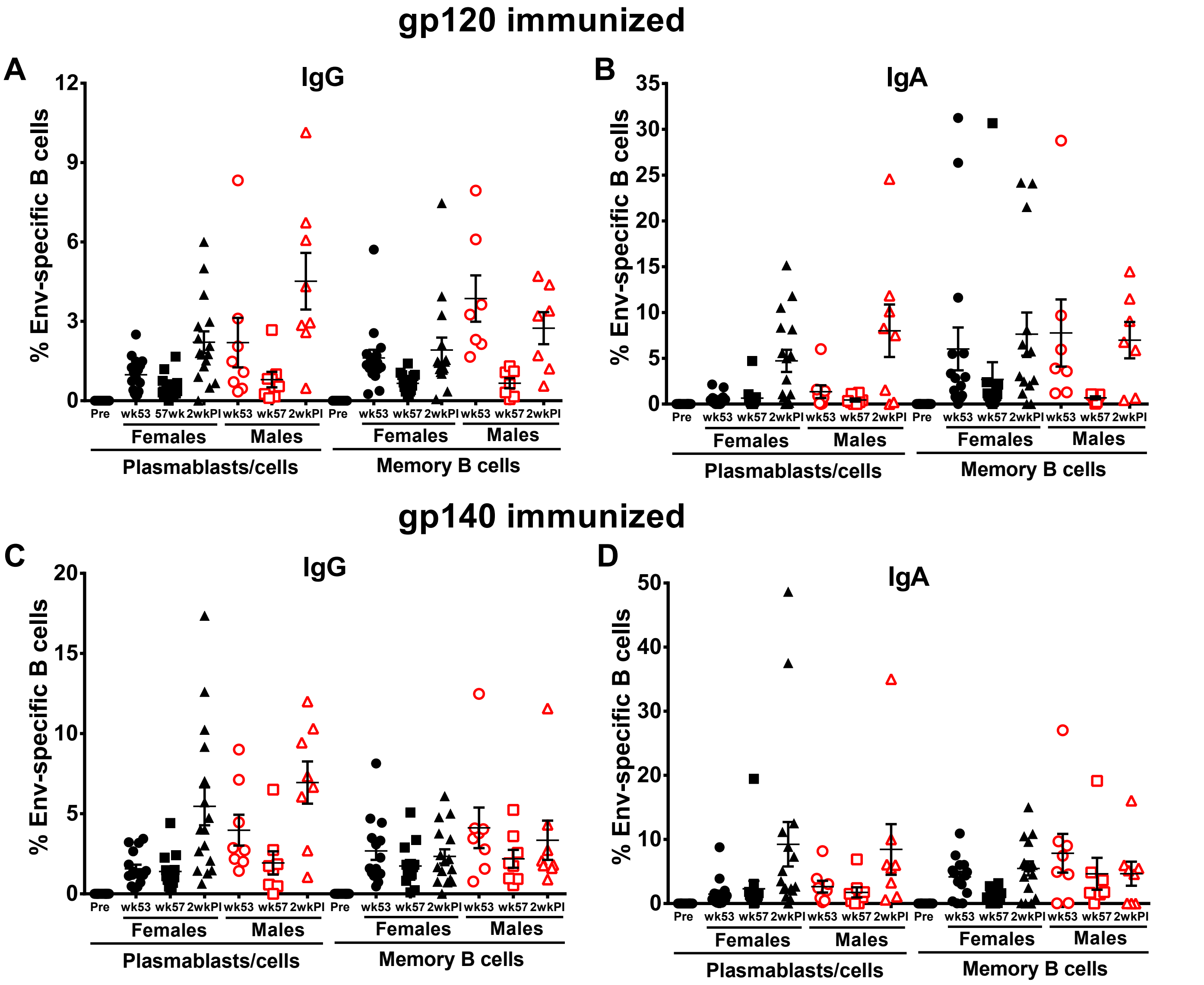

S9 Fig. Bone marrow Env-specific plasmablasts/plasma cells and memory B cells induced by vaccination in females and males. Env-specific plasmablasts/plasma cells and memory B cells secreting Env-specific IgG (A,C) and IgA (B,D) in gp120-(A,B) and gp140-(C,D) immunization groups by sex are shown. The gp120 group was tested against monomeric gp120 and gp140 group against oligomeric gp140 by ELISpot. Closed black symbols represent females and open red symbols represent males. Mean values ± SEM are shown.
