# Supplementary material for: Mucosal B Cells Are Associated with Delayed SIV Acquisition in Vaccinated Female but Not Male Rhesus Macaques Following SIVmac251 Rectal Challenge
Source: PLoS Pathog. 2015 Aug 12;11(8):e1005101. doi: 10.1371/journal.ppat.1005101 (PMC4534401; doi:10.1371/journal.ppat.1005101)
Supplement: S10 Fig — Env-specific IgA (A, C) and IgG (B, D) for gp120- (A, B) and gp140- (C, D) immunized females and males are shown over the course of immunization and 2wkpi. The gp120 group was tested against monomeric gp120 and the gp140 group against oligomeric gp140. All results were standardized to background control levels. Bars represent mean values ± SEM. (PDF) [file ppat.1005101.s010.pdf]

# gp120 immunized

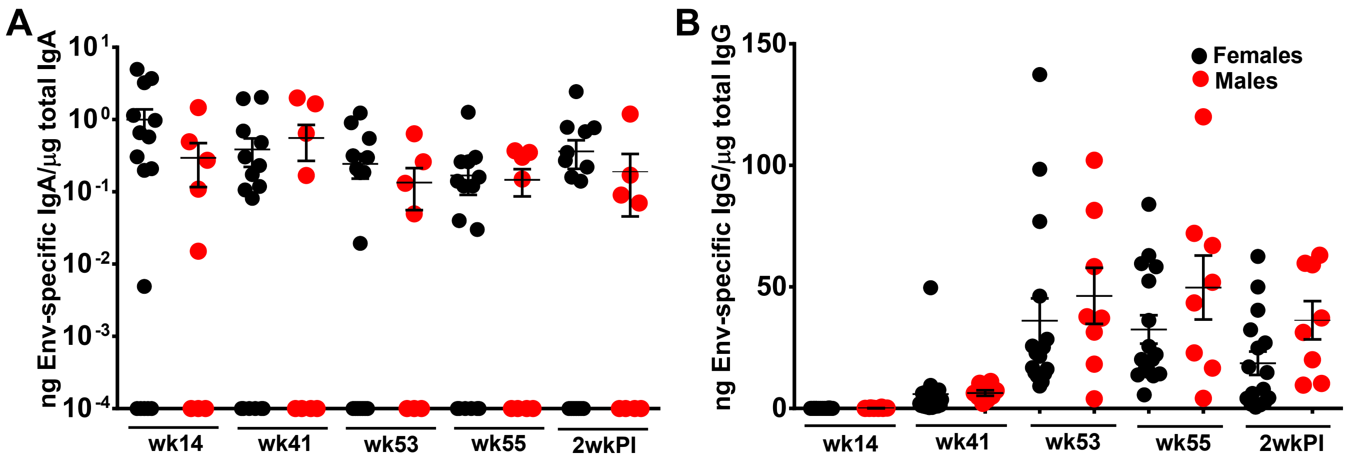


# gp140 immunized

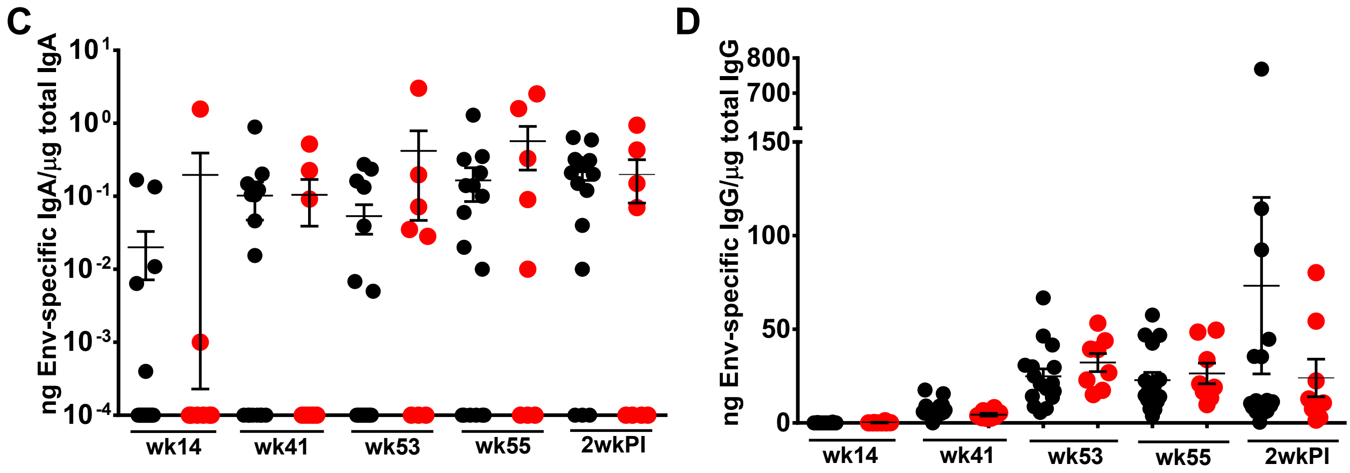


**S10 Fig. Env-specific IgA and IgG in rectal secretions of vaccinated females and males.** Env-specific IgA (A, C) and IgG (B, D) for gp120- (A, B) and gp140- (C, D) immunized females and males are shown over the course of immunization and 2wkpi. The gp120 group was tested against monomeric gp120 and the gp140 group against oligomeric gp140. All results were standardized to background control levels. Bars represent mean values ± SEM.
