# Supplementary material for: Mucosal B Cells Are Associated with Delayed SIV Acquisition in Vaccinated Female but Not Male Rhesus Macaques Following SIVmac251 Rectal Challenge
Source: PLoS Pathog. 2015 Aug 12;11(8):e1005101. doi: 10.1371/journal.ppat.1005101 (PMC4534401; doi:10.1371/journal.ppat.1005101)
Supplement: S12 Fig — No influence of rectal Env-specific IgG at wk 55 on the rate of acquisition in (A) all immunized macaques, (B) gp120-immunized or (C) gp140-immunized macaques, (D) all immunized females, (E) gp120- immunized or (F) gp140- immunized females, (G) all immunized males, (H) gp120-immunized or (I) gp140-immunized males. (PDF) [file ppat.1005101.s012.pdf]

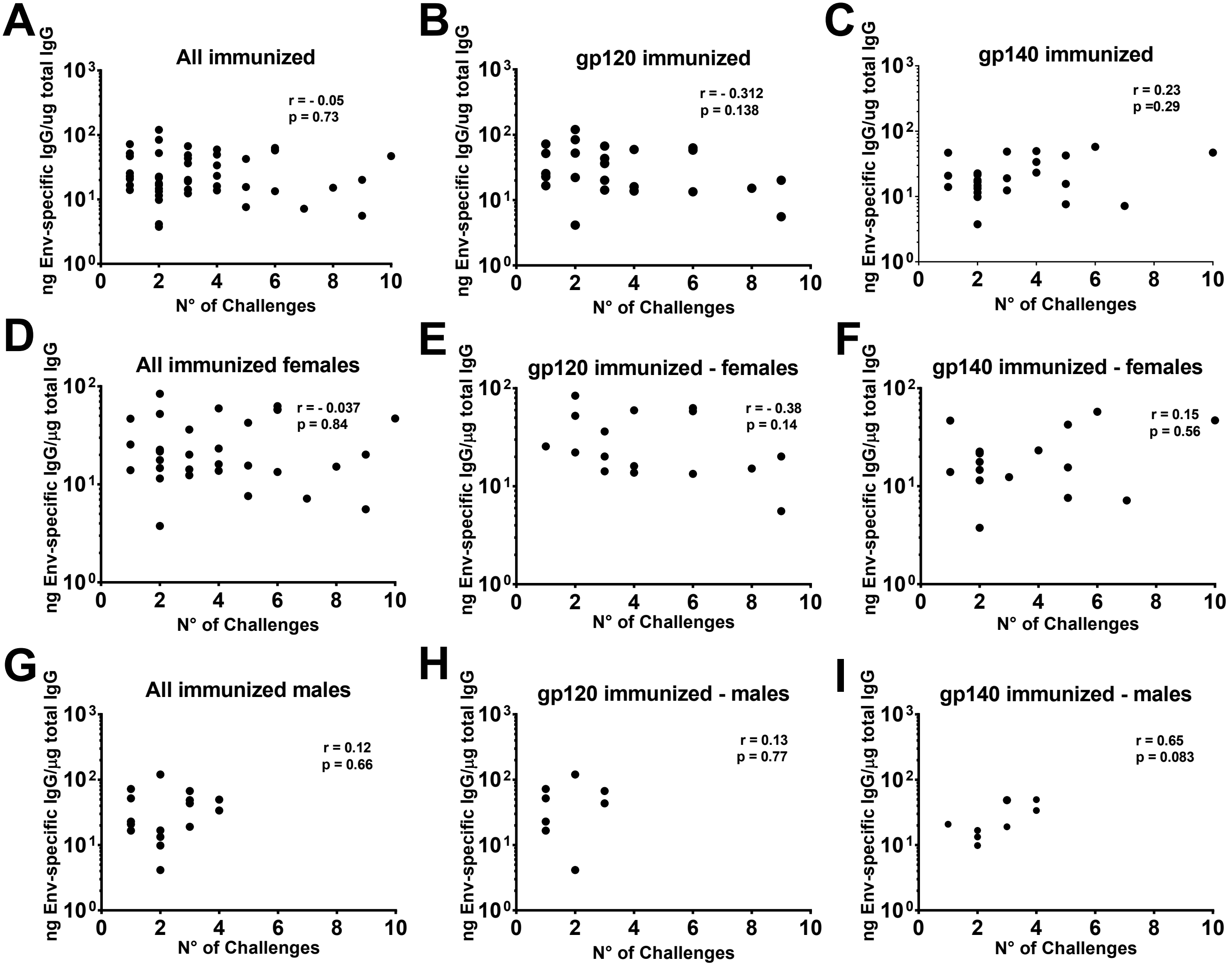

S12 Fig. Rectal Env- specific IgG not correlated with delayed SIV acquisition in immunized macaques. No influence of rectal Env- specific IgG at wk 55 on the rate of acquisition in (A) all immunized macaques, (B) gp120 - immunized or (C) gp140 - immunized macaques, (D) all immunized females, (E) gp120- immunized or (F) gp140- immunized females, (G) all immunized males, (H) gp120- immunized or (I) gp140- immunized males.
