# Supplementary material for: Mucosal B Cells Are Associated with Delayed SIV Acquisition in Vaccinated Female but Not Male Rhesus Macaques Following SIVmac251 Rectal Challenge
Source: PLoS Pathog. 2015 Aug 12;11(8):e1005101. doi: 10.1371/journal.ppat.1005101 (PMC4534401; doi:10.1371/journal.ppat.1005101)
Supplement: S13 Fig — No influence of rectal Env-specific IgA at wk 55 on the rate of infection in (A) all immunized males, (B) gp120-immunized males, and (C) gp140- immunized males. (PDF) [file ppat.1005101.s013.pdf]

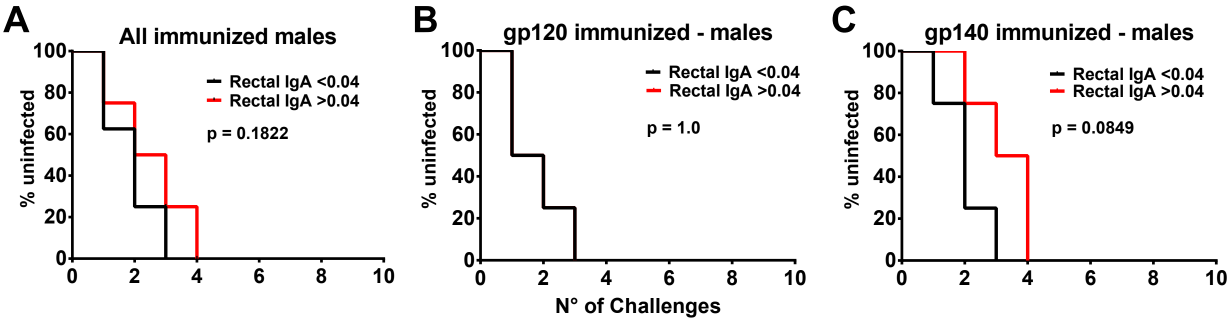


**S13 Fig. Rectal Env - specific IgA not correlated with delayed SIV acquisition in immunized males.** No influence of rectal Env-specific IgA at wk 55 on the rate of infection in (A) all immunized males, (B) gp120-immunized males, and (C) gp140- immunized males.
