# Supplementary material for: Mucosal B Cells Are Associated with Delayed SIV Acquisition in Vaccinated Female but Not Male Rhesus Macaques Following SIVmac251 Rectal Challenge
Source: PLoS Pathog. 2015 Aug 12;11(8):e1005101. doi: 10.1371/journal.ppat.1005101 (PMC4534401; doi:10.1371/journal.ppat.1005101)
Supplement: S14 Fig — (A) Example of flow cytometry staining for Env-specific memory B cells: Live CD2-CD14- cells from rectal pinches were gated for CD19+CD20+ B cells and then IgD+ B cells were excluded. The far right-hand plot shows Env-specific memory B cells in a vaccinated macaque and a control macaque using biotinylated gp120. (B) Example of flow cytometry staining for PB and PC. Live CD2-CD14- cells from rectal pinches were gated for CD19+CD20+ B cells and then IgD+ B cells were excluded. IgD- B cells were further gated for IRF4+CD138- and IRF4+ CD138+. PB (upper-right quadrant highlighted by the red box) are identified as CD19+ CD20+/-IgD-IRF4+ CD138- HLA-DR+Ki67+. PC (lower left quadrant highlighted by the red box) are identified as CD19+ CD20+/-IgD-IRF4+CD138+HLA-DR-Ki67-. (C) gp120-specific memory B cells quantified by flow cytometry correlate with frequency of Env-specific memory B cells secreting IgG + IgA by ELISpot in rectal biopsies from chronically SIV infected macaques. (PDF) [file ppat.1005101.s014.pdf]

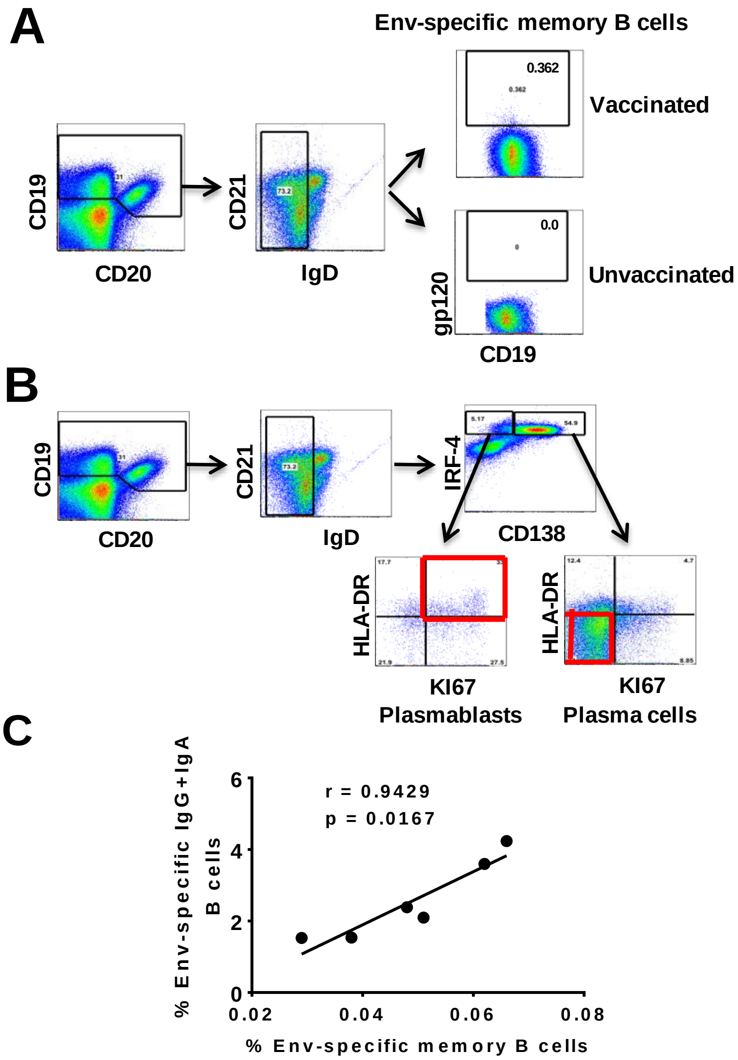

S14 Fig. Gating strategy for Env-specific memory B cells, plasmablasts and plasma cells in rectal tissue. (A) Example of flow cytometry staining for Env -specific memory B cells: Live CD2$^-$CD14$^-$ cells from rectal pinches were gated for CD19$^+$CD20$^+$ B cells and then IgD$^+$ B cells were excluded. The far right-hand plot shows Env-specific memory B cells in a vaccinated macaque and a control macaque using biotinylated gp120. (B) Example of flow cytometry staining for PB and PC. Live CD2$^-$CD14$^-$ cells from rectal pinches were gated for CD19$^+$CD20$^+$ B cells and then IgD$^+$ B cells were excluded. IgD$^-$ B cells were further gated for IRF4$^+$CD138$^-$ and IRF4$^+$ CD138$^+$. PB (upper - right quadrant highlighted by the red box) are identified as CD19$^+$ CD20$^{+/-}$IgD$^-$IRF4$^+$ CD138$^-$ HLA-DR$^+$Ki67$^+$. PC (lower left quadrant highlighted by the red box) are identified as CD19$^+$ CD20$^{+/-}$IgD$^-$IRF4$^+$CD138$^+$HLA-DR$^-$Ki67$^-$. (C) gp120–specific memory B cells quantified by flow cytometry correlate with frequency of Env-specific memory B cells secreting IgG + IgA by ELISpot in rectal biopsies from chronically SIV infected macaques.
