# Supplementary material for: Mucosal B Cells Are Associated with Delayed SIV Acquisition in Vaccinated Female but Not Male Rhesus Macaques Following SIVmac251 Rectal Challenge
Source: PLoS Pathog. 2015 Aug 12;11(8):e1005101. doi: 10.1371/journal.ppat.1005101 (PMC4534401; doi:10.1371/journal.ppat.1005101)
Supplement: S15 Fig — (A-C) Viral loads were recorded up to 40 wkpi. Female macaques are shown in black lines and males in red lines. A † marks macaques that were euthanized before 40 weeks of follow up. Macaque R663 in the gp140-immunized group resisted infection over 9 challenges, and is not shown. (PDF) [file ppat.1005101.s015.pdf]

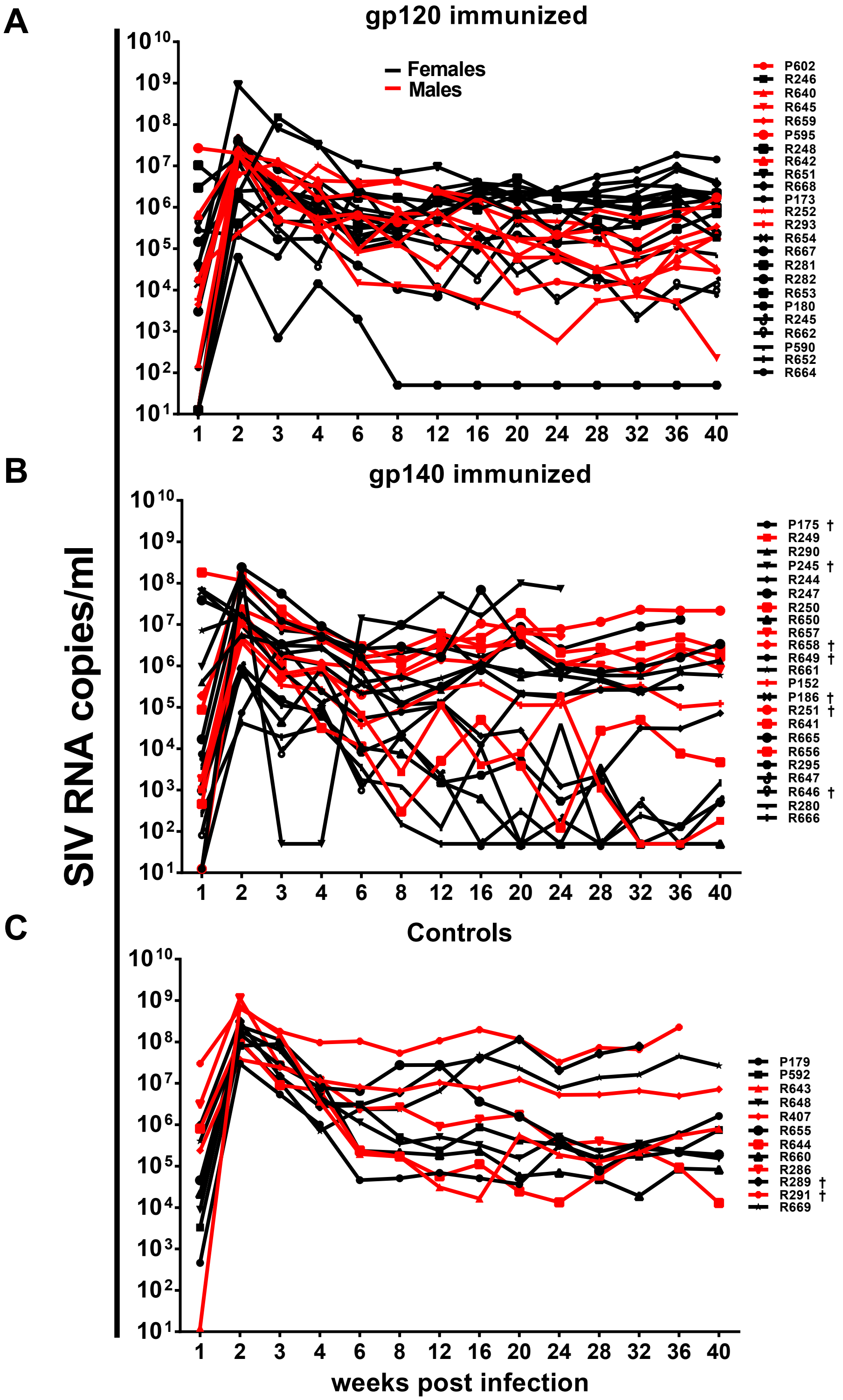

S15 Fig. Individual plasma viral loads for gp120 and gp140 immunized and control macaques. (A -C) Viral loads were recorded up to 40 wkpi. Female macaques are shown in black lines and males in red lines. A † marks macaques that were euthanized before 40 weeks of follow up. Macaque R663 in the gp140 - immunized group resisted infection over 9 challenges, and is not shown.
