# Supplementary material for: Mucosal B Cells Are Associated with Delayed SIV Acquisition in Vaccinated Female but Not Male Rhesus Macaques Following SIVmac251 Rectal Challenge
Source: PLoS Pathog. 2015 Aug 12;11(8):e1005101. doi: 10.1371/journal.ppat.1005101 (PMC4534401; doi:10.1371/journal.ppat.1005101)
Supplement: S16 Fig — Plasma viral loads (geometric mean) in historical and current controls (A) and in (B) combined control males and females. (PDF) [file ppat.1005101.s016.pdf]

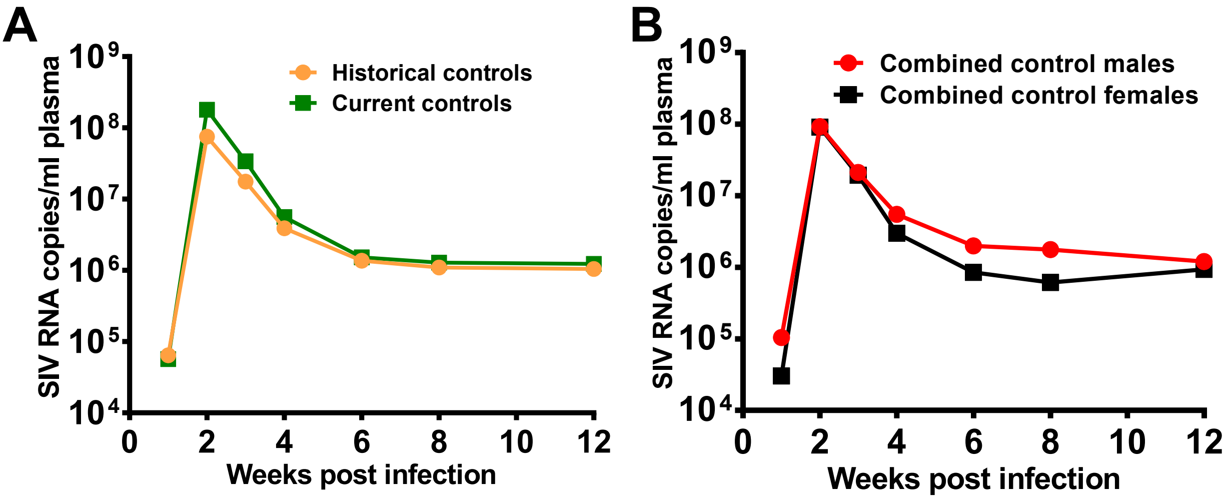

S16 Fig. Dynamics of plasma viral loads in SIV-infected historical and current control macaques. Plasma viral loads (geometric mean) in historical and current controls (A) and in (B) combined control males and females.
