# Supplementary material for: Mucosal B Cells Are Associated with Delayed SIV Acquisition in Vaccinated Female but Not Male Rhesus Macaques Following SIVmac251 Rectal Challenge
Source: PLoS Pathog. 2015 Aug 12;11(8):e1005101. doi: 10.1371/journal.ppat.1005101 (PMC4534401; doi:10.1371/journal.ppat.1005101)
Supplement: S17 Fig — Lack of correlation of serum (wk 53) ADCC against gp120 and gp140 targets expressed as 50% maximum killing titer from (A and B) gp120- and (C and D) gp140-immunized animals with peak viral load. No correlation of neutralizing antibody titers (wk 57) from (E) gp120- and (F) gp140-immunized animals with peak viral load. (PDF) [file ppat.1005101.s017.pdf]

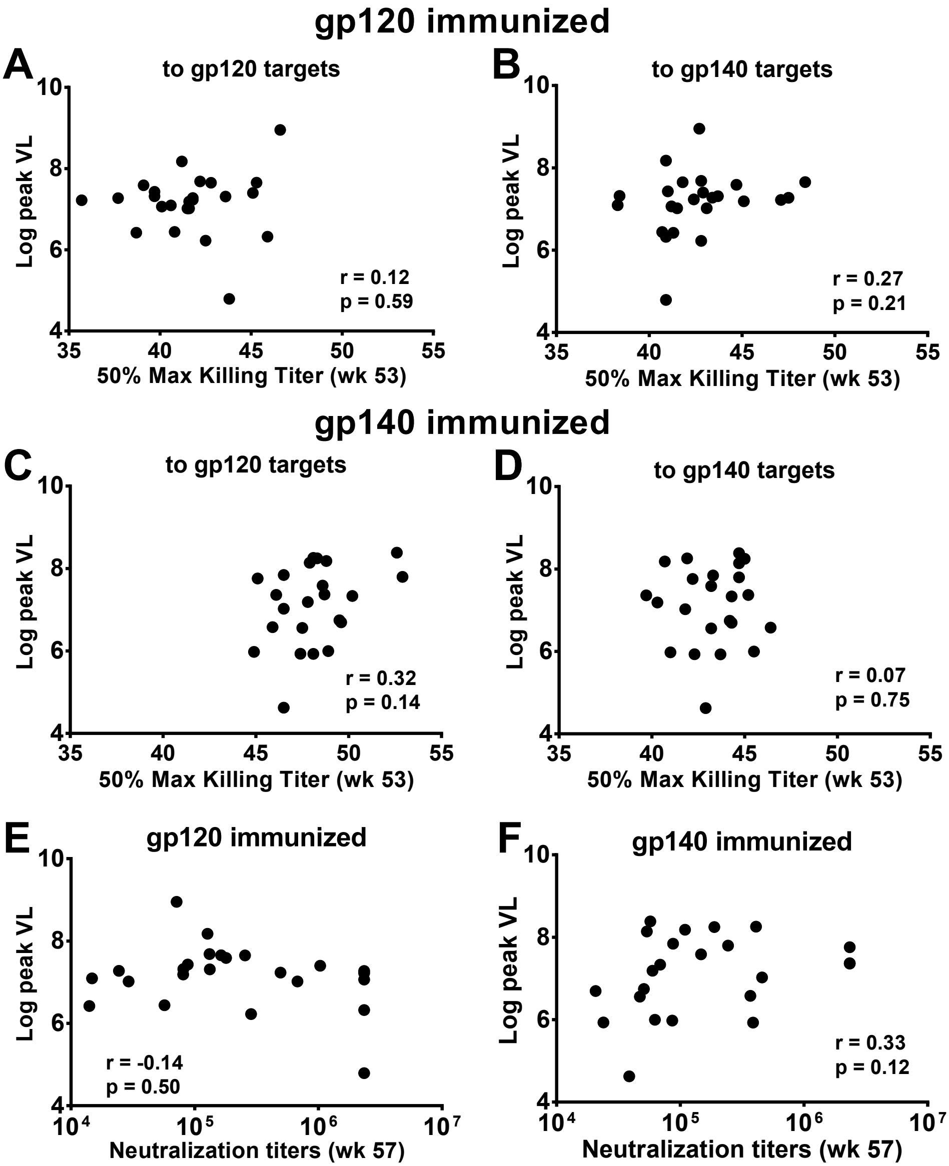

S17 Fig. No correlation of serum neutralizing and ADCC antibody activities with viremia control. Lack of correlation of serum (wk 53) ADCC against gp120 and gp140 targets expressed as 50% maximum killing titer from (A and B) gp120- and (C and D) gp140- immunized animals with peak viral load. No correlation of neutralizing antibody titers (wk 57) from (E) gp120- and (F) gp140- immunized animals with peak viral load.
