# Supplementary material for: Mucosal B Cells Are Associated with Delayed SIV Acquisition in Vaccinated Female but Not Male Rhesus Macaques Following SIVmac251 Rectal Challenge
Source: PLoS Pathog. 2015 Aug 12;11(8):e1005101. doi: 10.1371/journal.ppat.1005101 (PMC4534401; doi:10.1371/journal.ppat.1005101)
Supplement: S18 Fig — PBMC obtained 2wkpi from all macaque groups were assessed by intracellular cytokine staining for EnvsmH4-specific CD8+ T cells. Significant correlations were observed in all males with control of viremia (A) including reduced peak, median acute (weeks 1–6) and median chronic (over wk 8–24) viral loads. Similar correlations were not seen for all female macaques (B). CD8+ central and effector memory cells were summed and results for total memory (TM) CD8+ T cells are presented as the percent cytokine positive cells expressing IFN-γ, TNF-α and / or IL-2. Due to the large number of macaques, only half the macaques in each group were assessed for SIVsmH4 Env-specificity. (PDF) [file ppat.1005101.s018.pdf]

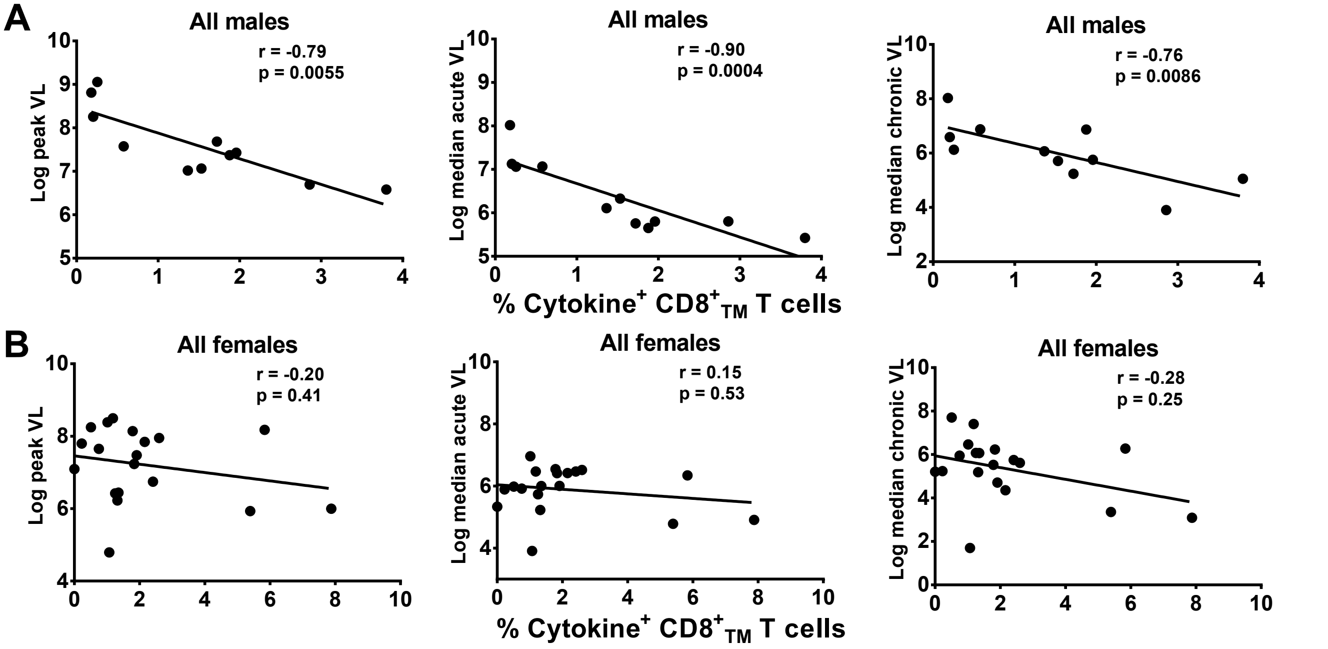

S18 Fig. SIV Env-specific CD8$^+$ T cell responses correlate with control of viremia in all males, but not in females. PBMC obtained 2wkpi from all macaque groups were assessed by intracellular cytokine staining for Env_{smH4}- specific CD8$^+$ T cells. Significant correlations were observed in all males with control of viremia (A) including reduced peak, median acute (weeks 1- 6) and median chronic (over wk 8 – 24) viral loads. Similar correlations were not seen for all female macaques (B). CD8$^+$ central and effector memory cells were summed and results for total memory (TM) CD8$^+$ T cells are presented as the percent cytokine positive cells expressing IFN-γ, TNF-α and / or IL-2. Due to the large number of macaques, only half the macaques in each group were assessed for SIV_{smH4} Env-specificity.
