# Supplementary material for: Mucosal B Cells Are Associated with Delayed SIV Acquisition in Vaccinated Female but Not Male Rhesus Macaques Following SIVmac251 Rectal Challenge
Source: PLoS Pathog. 2015 Aug 12;11(8):e1005101. doi: 10.1371/journal.ppat.1005101 (PMC4534401; doi:10.1371/journal.ppat.1005101)
Supplement: S19 Fig — Plasma viral loads (geometric mean) in females (A) and males (B) by immunization group. Absolute CD4+ T cell counts (mean values) in (C) females and (D) males by immunization group. *p < 0.05 for immunized males vs controls; p < 0.01 for immunized females vs controls. (PDF) [file ppat.1005101.s019.pdf]

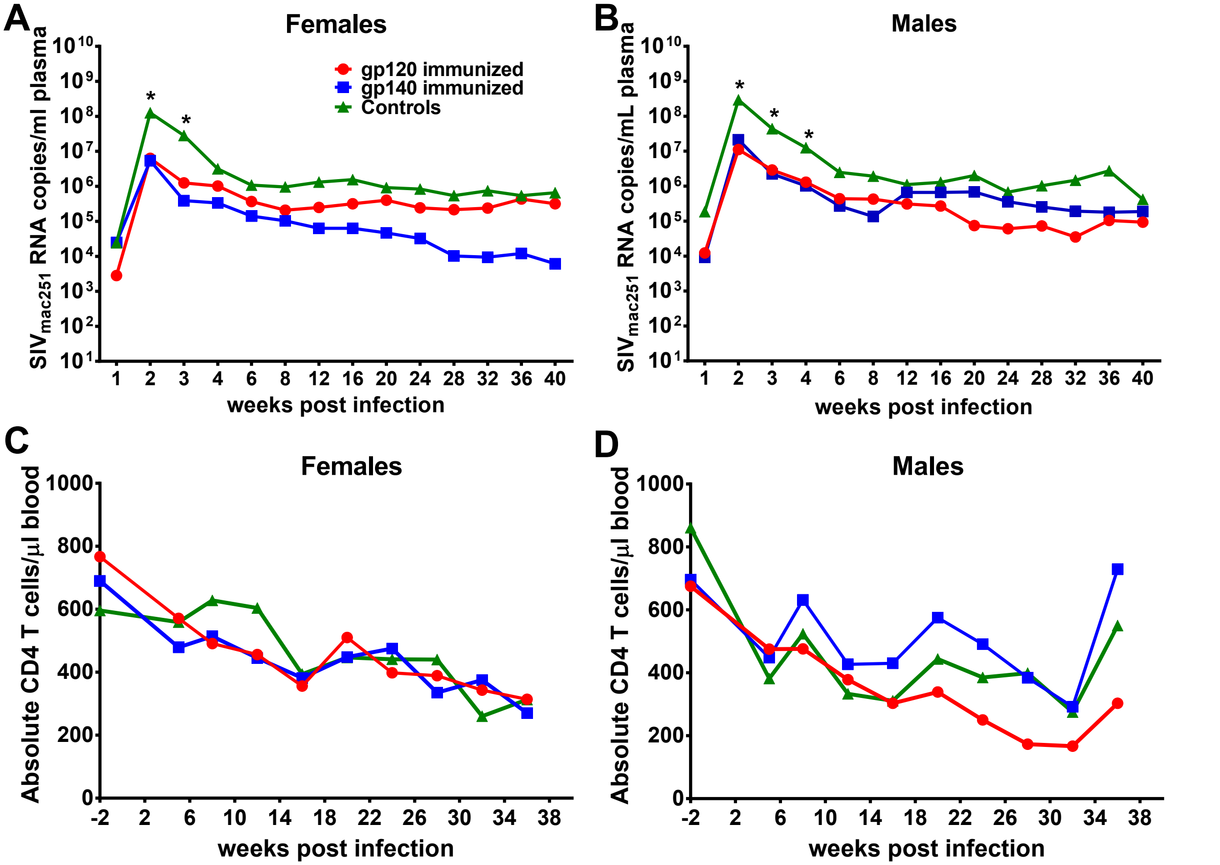

S19 Fig. Dynamics of plasma viral loads and CD4 counts in SIV - infected female and male rhesus macaques by immunization group. Plasma viral loads (geometric mean) in females (A) and males (B) by immunization group. Absolute $CD4^{+}$ T cell counts (mean values) in (C) females and (D) males by immunization group. *$p < 0.05$ for immunized males vs controls; $p < 0.01$ for immunized females vs controls.
